# ﻿An illustrated key to the lace bugs (Hemiptera, Heteroptera, Tingidae) from “Oriental Galapagos” (the Ogasawara Islands, Japan), with descriptions of three new species of the endemic genus *Omoplax* Horváth, 1912

**DOI:** 10.3897/zookeys.1250.160064

**Published:** 2025-08-29

**Authors:** Jun Souma

**Affiliations:** 1 Shirakami Research Center for Environmental Sciences, Faculty of Agriculture and Life Science, Hirosaki University, Aomori, Japan Hirosaki University Aomori Japan

**Keywords:** Allopatric distribution, East Asia, endemic taxa, host plant, Lauraceae, phytophagous insect, oceanic island, Oceanian Region

## Abstract

The lace bugs (Hemiptera, Heteroptera, Tingidae) from the Ogasawara Islands, Japan, which are known as “Oriental Galapagos”, are taxonomically revised. The following eight species belonging to the two endemic genera, *Acanthomoplax* Souma & Kamitani, 2021 (Tinginae, Tingini) or *Omoplax* Horváth, 1912 (Tinginae, Tingini) are recognized from the islands: *A. tomokunii* Souma & Kamitani, 2021, *O. desecta* (Horváth, 1912), *O. hisasuei***sp. nov.**, *O. inugusu***sp. nov.**, *O. karubei* Souma, 2022, *O. kobugashi***sp. nov.**, *O. majorcarinae* Guilbert, 2001, and *O. mukojimensis* Souma, 2022. In previous studies published in the 2020s, *O. inugusu***sp. nov.** and *O. kobugashi***sp. nov.** were misidentified as *O. majorcarinae*, while *O. majorcarinae*, re-diagnosed in the present study, was confused with *O. desecta*. Host plants for seven of the eight species, excluding *O. mukojimensis*, were revealed based on field and captive observations. Five of these seven species—*A. tomokunii*, *O. hisasuei***sp. nov.**, *O. inugusu***sp. nov.**, *O. kobugashi***sp. nov.**, and *O. majorcarinae*—feed on evergreen lauraceous trees. An illustrated key is also provided to identify all eight lace bug species from the Ogasawara Islands. Moreover, differences in host plants and distribution ranges of the eight endemic species are also discussed. Future research directions necessary for the conservation of endemic lace bugs are proposed.

## ﻿Introduction

The Ogasawara Islands, also known as “Oriental Galapagos”, comprise more than 100 oceanic islands located approximately 1,000 km south of the mainland of Tokyo Metropolis, Honshu, Japan, and they are the only part of Japan classified within the Oceanian Region (cf. Shimizu 2003; Karube and Takakuwa 2004). Despite their limited land area—the largest island, Chichijima Island, spans only 23.45 km^2^—the islands have an exceptionally high proportion of endemic species, with more than 90% of land snails, 30% of insects, and 40% of plants being endemic (Toyoda 1981; Kato 1992; Tomiyama 1992a; Shimizu 2003; Karube and Takakuwa 2004). In addition to the presence of numerous endemic species, the Ogasawara Islands are characterized by evolutionary phenomena unique to oceanic islands, including pronounced adaptive radiation among land snails and plants and the evolution of dioecy and woodiness in plants (Shimizu and Tabata 1991; Tomiyama 1992b; Shimizu 2003; Karube and Takakuwa 2004; Chiba 2010). Because of such a unique island ecosystem, the Ogasawara Islands were inscribed on the UNESCO World Natural Heritage site under the criteria (ix) “to be outstanding examples representing significant on-going ecological and biological processes in the evolution and development of terrestrial, fresh water, coastal and marine ecosystems and communities of plants and animals” (UNESCO World Heritage Centre 1992–2025; Ministry of the Environment 2025).

The Ogasawara Islands comprise three island groups: Mukojima, Chichijima, and Hahajima groups (Karube and Takakuwa 2004). In some insects, closely related endemic taxa are allopatrically distributed in specific island groups or islands (e.g., Yasunaga 2000; Morimoto et al. 2015); however, their mechanisms of speciation are poorly understood. Conducting an evolutionary biological study of endemic insect taxa with high species diversity can clarify the mechanisms of speciation caused by host shifts, geographic isolation, etc., thereby enhancing the value of the Ogasawara Islands as a World Natural Heritage site. However, in the Ogasawara Islands, insect species diversity, especially among small-sized taxa, remains poorly understood, with many undescribed species and unrecorded distributions. Therefore, further accumulation of basic knowledge, including taxonomic studies, is essential for conducting evolutionary biological studies on the endemic insects of the Ogasawara Islands.

Phytophagous insects, which are often studied in evolutionary biology, speciate via mechanisms such as host shifts and geographic isolation (e.g., Nakadai and Kawakita 2016; Hardy et al. 2022; Ward et al. 2022). Thus, evolutionary biological studies based on examples from other regions can be applied easily to the Ogasawara Islands. Lace bugs (Hemiptera, Heteroptera, Tingidae), known for their phytophagy and high host specificity (Schuh and Weirauch 2020), include numerous species endemic to oceanic islands (cf. Drake and Poor 1943; Froeschner 1976; Guilbert 2001), making them promising candidates for evolutionary biological studies on the endemic insects of the Ogasawara Islands. In the Ogasawara Islands, five lace bug species belonging to the two endemic genera, *Acanthomoplax* Souma & Kamitani, 2021 (Tinginae, Tingini) or *Omoplax* Horváth, 1912 (Tinginae, Tingini), have been recorded: *A. tomokunii* Souma & Kamitani, 2021, *O. desecta* (Horváth, 1912), *O. karubei* Souma, 2022, *O. majorcarinae* Guilbert, 2001, and *O. mukojimensis* Souma, 2022 (Shimamoto and Ishikawa 2023). Among them, *A. tomokunii*, *O. desecta*, and *O. majorcarinae* are distributed across several island groups, whereas *O. karubei* and *O. mukojimensis* are distributed only on Mukojima Island, belonging to Mukojima Group (Souma and Kamitani 2021; Souma 2022a). Previous taxonomic studies on lace bugs from the Ogasawara Islands have relied solely on specimens deposited in research institutions (e.g., Horváth 1912; Guilbert 2001; Souma and Kamitani 2021; Souma 2022a). Therefore, the host plants of all five species remain unknown. Field surveys conducted by researchers familiar with the collection methods of lace bugs are expected to not only discover additional undescribed species but also provide further basic knowledge, such as host plants and precise distribution ranges, which are crucial for inferring the mechanisms of speciation.

On the other hand, many endemic insects of the Ogasawara Islands, especially diurnal, non-poisonous, odorless, and small-sized taxa, have declined significantly because of predation by the invasive green anole, *Anolis carolinensis* Voight, 1832 (Squamata, Dactyloidae), and numerous species are threatened with extinction (Karube and Takakuwa 2004; Makihara et al. 2004; Karube 2005). In lace bugs, *O. desecta* has been confirmed in the stomach contents of green anole (Takahashi et al. 2014). However, owing to the limitations of identification based on body parts and the lack of taxonomic studies at the time (early 2010s), predation by the green anole of other endemic lace bug species may have been overlooked. Some endemic lace bug species, such as *A. tomokunii*, have fewer than ten recorded specimens (Souma and Kamitani 2021; Souma 2022a) and may be rare. Field surveys focusing on lace bugs in the Ogasawara Islands are required urgently to facilitate the conservation of these poorly known endemic insects.

In the fall of 2024 and spring of 2025, the author conducted a field survey on lace bugs in Chichijima and Hahajima groups, collecting numerous specimens and revealing host plants for all species occurring in both island groups. Additionally, a colleague of the author, Jinhyeong Park, surveyed Mukojima Island and revealed that *O. karubei* feeds on the leaves of Rhaphiolepis indica (L.) Lindl. var. tashiroi Hayata (Rosaceae). Other colleagues, who have also conducted fieldwork in the Ogasawara Islands, provided the author with many recently collected specimens of lace bugs. With the accumulation of a considerable number of individuals, the morphological characteristics of recently collected specimens were compared with those of specimens recorded in previous studies (Souma and Kamitani 2021; Souma 2022a). Taxonomic re-evaluation showed that the specimens recorded as *O. majorcarinae* in the 2020s consist of two morphological species: one feeds on the leaves of *Machilus kobu* Maxim. (Lauraceae) in Chichijima Group and the other feeds on the leaves of *M. boninensis* Koidz. on Hahajima Island, Hahajima Group. The two morphological species differ not only in the shape of the paranotum, which was previously considered intraspecific variation (Souma 2022a), but also in rostral length and hemelytral shape. Furthermore, two indeterminate species of the genus *Omoplax* feeding on the leaves of Neolitsea sericea (Blume) Koidz. var. aurata (Hayata) Hatus. (Lauraceae) were discovered in Chichijima Group and on Hahajima Island, respectively. The indeterminate species from Chichijima Group match well with the original description of *O. majorcarinae* (Guilbert 2001). Meanwhile, the specimens recorded as *O. majorcarinae* in the 2020s differ from the original description in body size, coloration, rostral length, and/or the shape of the paranotum and hemelytron, which have been previously considered intraspecific variations owing to the limited number of specimens at the time. These specimens, consisting of two morphological species feeding on *Machilus* species, differ in their morphological characteristics from all known lace bug species, and both were considered undescribed species. In addition, some specimens recorded as *O. desecta* from Ototojima Island, Chichijima Group, in the 2020s, correspond to *O. majorcarinae*. An indeterminate species feeding on N. sericea var. aurata on Hahajima Island also differs from all known lace bug species based on morphological characteristics and was considered an undescribed species. Moreover, field and captive observations have revealed that *A. tomokunii* feeds on the leaves of *M. kobu* on Ototojima Island, whereas *O. desecta* feeds on the leaves of R. indica var. tashiroi and *Calophyllum inophyllum* L. (Clusiaceae) in Chichijima and Hahajima groups. In conclusion, eight lace bug species belonging to two endemic genera were recognized in the Ogasawara Islands. Among them, seven each are folivorous species that feed on the abaxial side of the leaves, similar to many lace bugs (Schuh and Weirauch 2020). In addition, six each feed on a single tree species and are distributed in only a single island group.

In the present study, three new species are described: *O. hisasuei* sp. nov. from Hahajima Island, *O. inugusu* sp. nov. from Hahajima Island, and *O. kobugashi* sp. nov. from Chichijima Group. Furthermore, all known lace bug taxa from the Ogasawara Islands, including *O. majorcarinae*, are re-diagnosed, and the host plants for seven of the eight species, excluding *O. mukojimensis*, are newly recorded. An illustrated key for the identification of all eight lace bug species occurring in the Ogasawara Islands is provided. Finally, differences in host plants and distribution ranges among the eight species are discussed, and future research directions necessary for the conservation of endemic lace bugs are proposed.

## ﻿Materials and methods

Morphological characteristics of the specimens were observed, drawn, and measured under a stereoscopic microscope (SZX16; Olympus, Tokyo, Japan) equipped with an ocular grid. The specimens were photographed using a digital camera (EOS 90D; Canon, Tokyo, Japan) equipped with a zoom lens (18–35 mm F1.8 DC HSM; SIGMA, Kanagawa, Japan) and a digital microscope (Dino-Lite Premier M; Opto Science, Tokyo, Japan). Photographs of living individuals and host plants were taken using a compact digital camera (Tough TG-6; Olympus, Tokyo, Japan) and a smartphone (iPhone 14; Apple, California, USA), respectively. Image stacks of the specimens were processed using a Zerene Stacker (Zerene Systems, Washington, USA). All illustrations and photographs were processed using Adobe Photoshop 2024 ver.25.11 (Adobe Inc., San Jose, CA, USA). Morphological terms were assigned according to previous monographs (Drake and Davis 1960; Takeya 1962; Drake and Ruhoff 1965; Schuh and Weirauch 2020).

The specimens examined in the present study are deposited at the Entomological Laboratory, Faculty of Agriculture, Kyushu University, Fukuoka, Japan (**ELKU**); Kanagawa Prefectural Museum of Natural History, Kanagawa, Japan (**KPMNH**); National Museum of Nature and Science, Ibaraki, Japan (**NSMT**); Shirakami Research Center for Environmental Sciences, Faculty of Agriculture and Life Science, Hirosaki University, Aomori, Japan (**SIHU**); and Laboratory of Entomology, Faculty of Agriculture, Tokyo University of Agriculture, Kanagawa, Japan (**TUA**). All lace bug specimens from the Ogasawara Islands recorded in previous studies in the 2020s (Souma and Kamitani 2021; Souma 2022a) were re-examined in the present study. However, only specimens that were misidentified in the past are mentioned in the Results section.

Distribution maps of each species were created using the map data provided by the Geospatial Information Authority of Japan (GSI) (https://maps.gsi.go.jp) using the Adobe Photoshop software. The scientific names of the host plants were assigned according to Yonekura and Kajita (2003–2025).

## ﻿Systematics

### ﻿Genus Acanthomoplax

Taxon classificationAnimaliaHemipteraTingidae

Souma & Kamitani, 2021

A68334CB-1AD3-5DAE-8704-526486E3E785

Acanthomoplax Souma & Kamitani, 2021: 4. Type species by original designation: Acanthomoplax tomokunii Souma & Kamitani, 2021.

#### Note.

For detailed diagnostic characters of the genus, see Souma and Kamitani (2021) and Souma (2022a).

#### Remarks.

The monotypic genus *Acanthomoplax*, which is endemic to the Ogasawara Islands, Japan, comprises only *A. tomokunii* distributed in Chichijima and Hahajima groups (Souma and Kamitani 2021; Souma 2022a).

### ﻿ Acanthomoplax tomokunii

Taxon classificationAnimaliaHemipteraTingidae

Souma & Kamitani, 2021

728BA545-5186-5341-8757-B14F20C9E19A

Figs 1A, 2A, 3A, 4A, 5A, 6A, 7A, 8A, 9A, 10A, 11A, 12A, 13A, 14A, 15A–E

Acanthomoplax tomokunii Souma & Kamitani, 2021: 6. Holotype: ♀; type locality: Japan • Ogasawara Isls., Hahajima I., Mt. Chibusayama [= Ogasawara Islands, Hahajima Group, Hahajima Island, Mt. Chibusa]; ELKU.

#### References.

Souma (2022a: 125) (distribution); Shimamoto and Ishikawa (2023: 93) (catalog); Souma (2023: 9) (monograph).

**Figure 1.**
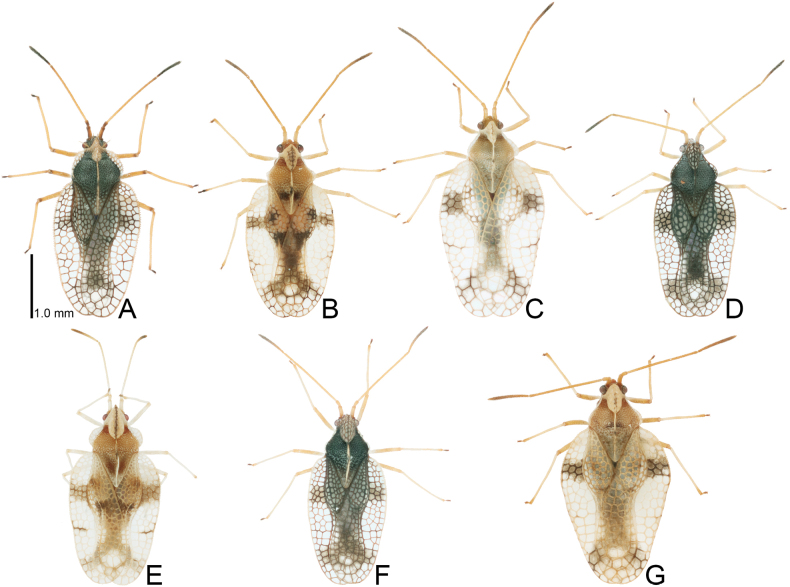
Males of seven tingid species endemic to the Ogasawara Islands, Japan, dorsal view. A. *Acanthomoplax tomokunii*; B. *Omoplax desecta*; C. *O. hisasuei* sp. nov.; D. *O. inugusu* sp. nov.; E. *O. karubei*; F. *O. kobugashi* sp. nov.; G. *O. majorcarinae*.

#### Material examined.

***Non-types***, Japan • 2 ♂♂ 1 ♀; Ogasawara Isls., Ototojima Is., Kurohama–Ichinotani; *Machilus kobu*; 5 Oct. 2024; J. Souma leg.; three third or fourth instar nymphs developed into adults by 15 Oct. 2024 by feeding on *Machilus kobu* in captivity; SIHU • 1 ♀; same collection data as for preceding; a single egg has developed into a second instar nymph until 17 Oct. 2024 by feeding on *Machilus kobu* in captivity, and a second instar nymph developed into an adult by 30 Oct. 2024 by feeding on *Machilus thunbergii* in captivity; SIHU.

#### Diagnosis.

*Acanthomoplax tomokunii* is recognized among other lace bug species based on diagnostic characters mentioned in previous studies (Souma and Kamitani 2021; Souma 2022a) and can be distinguished from the seven other lace bug species occurring in the Ogasawara Islands based on a combination of the following characteristics: pair of frontal spines reaching beyond apex of clypeus (Figs 3A, 4A, 5A, 6A, 14A); median spine reaching beyond bases of frontal spines; pair of occipital spines reaching beyond anterior margin of compound eye; hood medially with robust denticles throughout its length; median carina of pronotum with robust denticles throughout its length; paranotum narrowed posteriad; outer margin of paranotum with robust denticles throughout its length; Sc (subcosta) vein of hemelytron with robust denticles throughout its length; and R+M (fused radius and media) vein of hemelytron with robust denticles throughout its length (Figs 7A, 8A, 9A, 10A).

#### Remarks.

**Figure 2.**
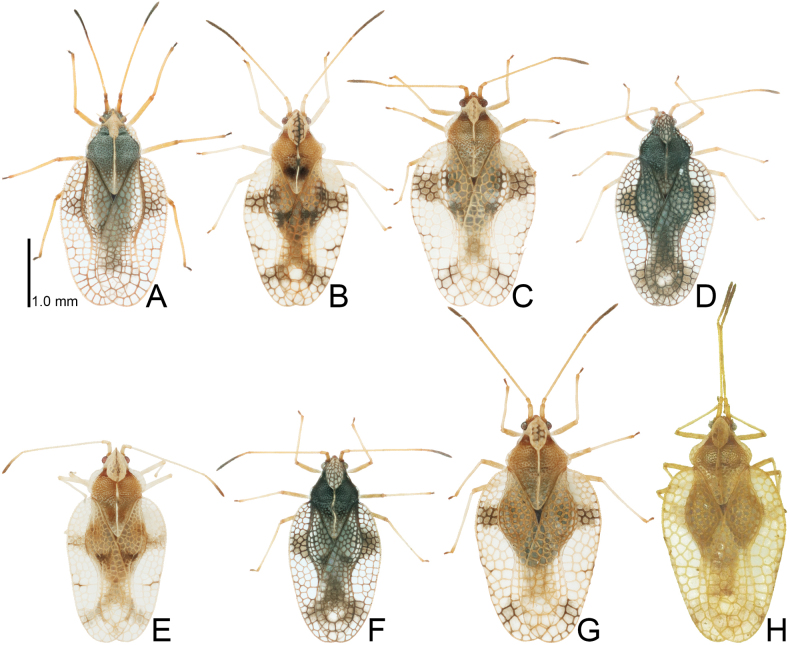
Females of eight tingid species endemic to the Ogasawara Islands, Japan, dorsal view. A. *Acanthomoplax tomokunii*; B. *Omoplax desecta*; C. *O. hisasuei* sp. nov.; D. *O. inugusu* sp. nov.; E. *O. karubei*; F. *O. kobugashi* sp. nov.; G. *O. majorcarinae*; H. *O. mukojimensis*.

Segmental oligomery of the antenna was confirmed in *Acanthomoplax tomokunii*, and one examined specimen lacks the left antennal segment IV (Fig. 2A), as reported in many lace bugs (Štusák and Stehlík 1978).

#### Distribution.

Japan: Ogasawara Islands: Chichijima Group (Anijima Island, Ototojima Island), Hahajima Group (Hahajima Island) (Fig. 18) (Souma and Kamitani 2021; Souma 2022a). *Acanthomoplax tomokunii*, which is endemic to the Ogasawara Islands, was confirmed in Ototojima Island during the survey in 2024 but has not been collected in Anijima and Hahajima islands since 2014 and 1999, respectively, despite extensive field surveys by numerous investigators (cf. Souma and Kamitani 2021; Souma 2022a).

#### Host plant.

*Machilus kobu* (Lauraceae) (Fig. 17A), which is also known as “Kobugashi” and is endemic to the Ogasawara Islands (Government of Japan 2010), was confirmed as a host plant for *Acanthomoplax tomokunii* through field and captive observations of adults and nymphs. In captivity, *A. tomokunii* fed on *M. thunbergii* Siebold & Zucc., “Tabunoki” (Fig. 17B), which is not distributed in the Ogasawara Islands (Tanaka and Matsui 2007–2025), and a single second instar nymph developed normally to an adult in at least 13 days, suggesting the possibility of rearing this species in captivity using closely related species of *M. kobu*, which are not found in the native distribution area of *A. tomokunii*. However, to the best of the author’s knowledge, *A. tomokunii* feeds only on *M. kobu* in the field and appears to be monophagous. Additionally, the single individual in the present study was reared on *M. kobu* to the second instar nymph. Therefore, it is unclear whether rearing from oviposition to emergence can be achieved successfully only by feeding on *M. thunbergii*.

#### Bionomics.

*Acanthomoplax tomokunii* inhabits an evergreen broad-leaved forest with a subtropical climate in the Ogasawara Islands (Souma and Kamitani 2021), and sucks sap on the abaxial side of the leaves of *M. kobu*, causing irregular yellowing on the adaxial side (Fig. 17A). Adults were collected in March and April, and from June to August (Souma and Kamitani 2021; Souma 2022a); nymphs were collected in October.

### ﻿Genus Omoplax

Taxon classificationAnimaliaHemipteraTingidae

Horváth, 1912

C031F63A-3422-54E4-842A-A29F93B04299

Omoplax Horváth, 1912: 336 (as subgenus of Stephanitis Stål, 1873; upgraded by Takeya 1962: 74). Type species by monotypy: Stephanitis (Omoplax) desecta Horváth, 1912.

#### Note.

For detailed diagnostic characters of the genus, see Souma and Kamitani (2021) and Souma (2022a).

#### Remarks.

The genus *Omoplax*, which is endemic to the Ogasawara Islands, Japan, previously comprised four species (Souma 2022a); however, in the present study, the author describes three new species. The following seven *Omoplax* species are currently recognized: *O. desecta*, *O. hisasuei* sp. nov., *O. inugusu* sp. nov., *O. karubei*, *O. kobugashi* sp. nov., *O. majorcarinae*, and *O. mukojimensis*.

### ﻿ Omoplax desecta

Taxon classificationAnimaliaHemipteraTingidae

(Horváth, 1912)

5B505D71-4ACA-5F73-B90C-9255F47A87CA

Figs 1B, 2B, 3B, 4B, 5B, 6B, 7B, 8B, 9B, 10B, 11B, 12B, 13B, 14B, 15F–H

Stephanitis (Omoplax) desecta Horváth, 1912: 337. Syntype(s): ♀; type locality: Japan • Ogasawara [= Ogasawara Islands]; Laboratory of Systematic Entomology, Faculty of Agriculture, Hokkaido University, Sapporo, Japan (Tomokuni 1994: 844; Souma 2022a: 125).
Omoplax desecta : Takeya (1962: 74) (new combination).

#### References.

Drake (1948: 55) (checklist: genus); Takeya (1951: 14) (checklist: eastern Asia); Drake and Maa (1953: 100) (checklist: genus); Drake (1956: 116) (distribution); Drake and Ruhoff (1960: 83) (catalog); Drake and Ruhoff (1965: 336) (catalog); Nakane (1970: 28) (checklist: Ogasawara Islands); Miyamoto and Yasunaga (1989: 167) (checklist: Japan); Nishimura and Arai (1989: 38) (distribution); Kato (1992: 79) (checklist: Ogasawara Islands); Yasunaga et al. (1993: 179) (monograph); Tomokuni (1994: 844) (type material); Yasunaga (1995: 20) (illustration); Péricart and Golub (1996: 51) (checklist: Palearctic); Miyano (1998: 8) (distribution); Guilbert (2001: 551) (distribution); Karube and Takakuwa (2004: 83) (illustration); Ohbayashi et al. (2004: 32) (checklist: Ogasawara Islands); Government of Japan (2010: 208) (checklist: Ogasawara Islands); Yamada and Tomokuni (2012: 198) (monograph); Takahashi et al. (2014: 163) (natural enemy); Yamada and Ishikawa (2016: 432) (checklist: Japan); Souma and Kamitani (2021: 8) (distribution: part); Souma (2022a: 125) (distribution: part); Shimamoto and Ishikawa (2023: 93) (catalog: part); Souma (2023: 9) (monograph). Several studies conducted before the 1980s recorded this species as Stephanitis (Omoplax) desecta (Drake 1948; Takeya 1951; Drake and Maa 1953; Drake 1956; Drake and Ruhoff 1960, 1965; Nakane 1970; Nishimura and Arai 1989).

#### Material examined.

***Non-types***, Japan • 1 ♂; Ogasawara Isls, Chichijima Is.; 6 May 1974; Y. Hori leg.; NSMT • 1 ♀; same locality data as for preceding; Rhaphiolepis indica var. tashiroi; 20 Mar. 2001; G. Tokihiro; NSMT • 1 ♀; Ogasawara Isls, Hahajima Is., Higashiko; 5 Jun. 1976; T. Nakane leg.; NSMT • 1 ♀; Ogasawara Isls, Hahajima Is., Mt. Chibusa; 8 Jun. 1976; T. Nakane leg.; NSMT • 1 ♀; same locality data as for preceding; 6 Jul. 1997; T. Kishimoto leg; NSMT • 2 ♀♀; same locality data as for preceding; 7 Jul. 1997; K. Matsumoto leg; NSMT • 1 ♀; same locality data as for preceding; 27 May 2022; T. Yoshida leg.; SIHU • 2 ♂♂ 5 ♀♀; same locality data as for preceding; Rhaphiolepis indica var. tashiroi; 2 Oct. 2024; J. Souma; SIHU • 1 ♂; Ogasawara Isls, Chichijima Is., Mt. Mikazuki; 12 Jun. 1976; T. Nakane leg.; NSMT • 23 ♂♂ 18 ♀♀ 1 fifth instar nymph; same locality data as for preceding; 18 Apr. 1997; K. Matsumoto leg.; NSMT • 1 ♀; same locality and date data as for preceding; T. Kishimoto leg.; NSMT • 1 ♂ 3 ♀♀; same locality data as for preceding; 23 Apr. 1997; K. Matsumoto leg.; NSMT • 2 ♂♂ 6 ♀♀; same locality data as for preceding; 17 May 2024; Y. Hisasue leg.; SIHU • 1 ♂ 1 ♀ 1 fifth instar nymph; same locality data as for preceding; Rhaphiolepis indica var. tashiroi; 21 Sep. 2024; J. Souma; SIHU • 2 ♀♀; same locality, host plant, and collector data as for preceding; 22 Sep. 2024; SIHU • 2 ♀♀; same locality data as for preceding; 25 Oct. 2024; Y. Hisasue; SIHU • 1 ♀; same locality and collector data as for preceding; 5 Nov. 2024; SIHU • 1 ♀; same locality and collector data as for preceding; 10 Jan. 2025; SIHU • 3 ♂♂ 4 ♀♀; same locality and collector data as for preceding; 15 May 2025; SIHU • 1 ♂ 1 ♀; Ogasawara Isls, Chichijima Is., Mt. Shigure; 8 Dec. 1977; M. Tomokuni leg.; NSMT • 1 ♀; Ogasawara Isls, Chichijima Is., Mt. Takayama; 30 Jul. 1996; K. Matsumoto leg.; NSMT• 2 ♀♀; Ogasawara Isls, Hahajima Is., Kuwanoki-zawa; 4 Aug. 1996; K. Matsumoto leg.; NSMT • 1 ♂; Ogasawara Isls, Chichijima Is., Higashidaira; 15 Apr. 1997; K. Matsumoto leg.; NSMT • 14 ♂♂ 8 ♀♀ 3 fifth instar nymphs 1 fourth instar nymph; Ogasawara Isls, Chichijima Is., Buta Beach; 16 Apr. 1997; K. Matsumoto leg.; NSMT • 7 ♂♂ 4 ♀♀; same locality and date data as for preceding; T. Kishimoto leg.; NSMT • 1 ♀; Ogasawara Isls, Chichijima Is., Nagatani–Mt. Tsutsuji; 17 Apr. 1997; K. Matsumoto leg; NSMT • 18 ♂♂ 14 ♀♀; Ogasawara Isls, Hahajima Is., Minamizaki; 19 Apr. 1997; K. Matsumoto leg; NSMT • 1 ♂; same locality data as for preceding; 4 Jul. 1997; T. Kishimoto leg; NSMT • 1 ♂; same locality data as for preceding; 15 Jul. 2024; Y. Uehara leg.; SIHU • 1 ♀; same locality data as for preceding; Rhaphiolepis indica var. tashiroi; 27 Sep. 2024; J. Souma; SIHU • 1 ♂ 3 ♀♀; Ogasawara Isls, Hahajima Is., Nakanotaira–Minamizaki; 19 Apr. 1997; K. Matsumoto leg; NSMT • 1 ♂; Ogasawara Isls, Hahajima Is., Mt. Mikazuki (non-existent place name: mislabeling?); 19 Apr. 1997; K. Matsumoto leg; NSMT • 1 ♂ 1 ♀; Ogasawara Isls, Hahajima Is., Komoridani; 20 Apr. 1997; K. Matsumoto leg; NSMT • 1 ♂ 1 ♀; Ogasawara Isls, Hahajima Is., Mt. Yakeyama; 20 Apr. 1997; K. Matsumoto leg; NSMT • 3 ♂♂; Ogasawara Isls, Hahajima Is., Oki-mura; 20 Apr. 1997; K. Matsumoto leg; NSMT • 1 ♀; same locality and date data as for preceding; T. Kishimoto leg; NSMT • 1 ♀; Ogasawara Isls, Chichijima Is., Minamifukurozawa; 23 Apr. 1997; K. Matsumoto leg; NSMT • 3 ♀♀ 1 fifth instar nymph; same locality data as for preceding; 23 Mar. 2024; Y. Hisasue leg.; SIHU • 1 ♀; Ogasawara Isls, Chichijima Is., Ichinotani; 26 Apr. 1997; K. Matsumoto leg; NSMT • 1 ♂; Ogasawara Isls, Ototojima Is., Kurohama; 27 Apr. 1997; K. Matsumoto leg; NSMT • 2 ♂♂; Ogasawara Isls, Hahajima Is., Nishiura; 4 Jul. 1997; K. Matsumoto leg; NSMT • 1 ♀; Ogasawara Isls, Hahajima Is., Mt. Kuwanoki; 4 Jul. 1997; T. Kishimoto leg; NSMT • 1 ♂ 1 ♀; Ogasawara Isls, Hahajima Is., Tamagawa Dam; 5 Jul. 1997; K. Matsumoto leg.; NSMT • 1 ♀; same locality data as for preceding; 14 Jul. 2024; Y. Uehara leg.; SIHU • 1 ♀; Ogasawara Isls, Chichijima Is., Mt. Tsutsuji; 10 Jul. 1997; T. Kishimoto leg.; NSMT • 1 ♂; Ogasawara Isls, Ototojima Is., Ainosawa; 1 Jul. 2021; T. Matsumoto & S. Shimamoto leg.; SIHU• 6 ♂♂ 4 ♀♀; same locality data as for preceding; 3–4 Jul. 2024; N. Tsuji leg.; SIHU • 2 ♀♀; Ogasawara Isls, Anijima Is., Central Plateau; 1 Jul. 2021; T. Matsumoto & S. Shimamoto leg.; SIHU • 1 ♂; same locality and collector data as for preceding; 2 Jul. 2021; SIHU • 1 ♀; Ogasawara Isls, Anijima Is., Mt. Togari; 29 Jul. 2021; T. Matsumoto & S. Shimamoto leg.; SIHU • 1 ♂; Ogasawara Isls, Hahajima Is., Igumadani, Sekimon; alt. 370 m; 26 May 2022; T. Yoshida leg.; SIHU • 2 ♂♂; Ogasawara Isls, Chichijima Is., Susaki; 21 May 2023; Y. Hisasue leg.; SIHU • 1 ♀; Ogasawara Isls, Chichijima Is., Mt. One; 4 Feb. 2024; Y. Hisasue leg.; SIHU • 2 ♂♂ 1 ♀; Ogasawara Isls, Meijima Is.; 14 Jun. 2024; N. Tsuji leg.; SIHU • 1 ♂; Ogasawara Isls, Hahajima Is., Nishidai; 17 Jun. 2024; N. Tsuji leg.; SIHU • 1 ♂ 5 ♀♀; Ogasawara Isls, Hahajima Is., Shizukazawa; 20 Jun. 2024; Y. Hisasue leg.; SIHU • 7 ♂♂ 6 ♀♀; Ogasawara Isls, Chichijima Is., Mt. Ogami; alt. 10–90 m; 11 Jul. 2024; Y. Uehara leg.; SIHU • 1 ♀; Ogasawara Isls, Ototojima Is., Mt. Sokuryogatake; 12 Jul. 2024; Y. Uehara leg.; SIHU • 1 ♂ 1 ♀; Ogasawara Isls., Anijima Is., Mansaku–Kanaimisaki; 12 Jul. 2024; N. Tsuji leg.; SIHU • 1 ♀; Ogasawara Isls, Hahajima Is., Nijuccho Pass; alt. 160 m; 15 Jul. 2024; Y. Uehara leg.; SIHU • 5 ♂♂ 1 ♀; Ogasawara Isls, Mukohjima Is.; alt. 0–90 m; 16 Jul. 2024; Y. Uehara leg.; SIHU • 1 ♀; Ogasawara Isls, Chichijima Is., Mt. Tsuitate; 19 Jul. 2024; Y. Hisasue leg.; SIHU • 1 ♀; Ogasawara Isls, Chichijima Is., Hatsuneura; 28 Jul. 2024; N. Tsuji leg.; SIHU • 1 ♂ 1 ♀; Ogasawara Isls, Chichijima Is., Higashi-machi; *Calophyllum inophyllum*; 21 Sep. 2024; J. Souma leg.; SIHU • 1 ♀; same locality, host plant, and collector data as for preceding; 8 Oct. 2024; SIHU • 1 ♀; Ogasawara Isls, Chichijima Is., Mt. Yoake; Rhaphiolepis indica var. tashiroi; 22 Sep. 2024; J. Souma leg.; SIHU • 1 ♂ 1 ♀; Ogasawara Isls., Ototojima Is., Kurohama–Ichinotani; Rhaphiolepis indica var. tashiroi; 23 Sep. 2024; J. Souma leg.; SIHU • 3 ♂♂ 1 ♀; Ogasawara Isls., Anijima Is., Tamana Beach–Mt. Mikaeri; Rhaphiolepis indica var. tashiroi; 24 Sep. 2024; J. Souma leg.; SIHU • 1 ♀; Ogasawara Isls, Chichijima Is., Ogamiyama Park; Rhaphiolepis indica var. tashiroi; 25 Sep. 2024; J. Souma leg.; SIHU • 1 ♂; Ogasawara Isls, Chichijima Is., John Beach; *Calophyllum inophyllum*; 7 Oct. 2024; J. Souma leg.; SIHU • 2 ♀♀; Ogasawara Isls, Nishijima Is.; 17 Nov. 2024; N. Tsuji leg.; SIHU • 1 ♂; Ogasawara Isls, Chichijima Is., Oku-mura; 10 Dec. 2024; Y. Hisasue leg.; SIHU • 1 ♀; Ogasawara Isls, Hahajima Is., Hyogidaira; Rhaphiolepis indica var. tashiroi; 9 Mar. 2025; J. Souma leg.; SIHU • 1 ♀; same locality, host plant, and collector data as for preceding; 11 Mar. 2025; SIHU • 1 ♂; Ogasawara Isls, Chichijima Is., Kominato Beach; 25 Apr. 2025; Y. Hisasue leg.; SIHU • 1 ♀; Ogasawara Isls, Chichijima Is., Mt. Sakaiura; 27 Apr. 2025; Y. Hisasue leg.; SIHU • 1 ♂; Ogasawara Isls, Chichijima Is., Mulberry Bay; 29 Apr. 2025; Y. Hisasue leg.; SIHU • 1 ♀; Ogasawara Isls, Hahajima Is., Nishiura; 4 May 2025; Y. Hisasue leg.; SIHU • 1 ♂ 4 ♀♀; Ogasawara Isls, Ototojima Is., Shikahama–Mt. Hirone; 27 May 2025; S. Shimamoto leg.; SIHU • 3 ♂♂ 5 ♀♀; Ogasawara Isls, Ototojima Is., Kohama; 2 Jun. 2025; Y. Hisasue leg.; SIHU. The four nymphs recorded above are in poor condition and are thus not described in the present study.

**Figure 3.**
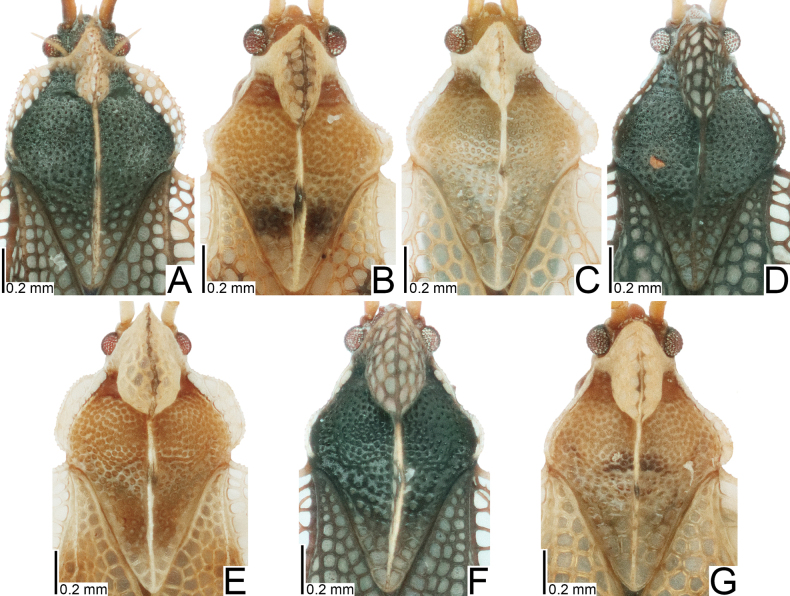
Male pronota of seven tingid species endemic to the Ogasawara Islands, Japan, dorsal view. A. *Acanthomoplax tomokunii*; B. *Omoplax desecta*; C. *O. hisasuei* sp. nov.; D. *O. inugusu* sp. nov.; E. *O. karubei*; F. *O. kobugashi* sp. nov.; G. *O. majorcarinae*.

#### Diagnosis.

**Figure 4.**
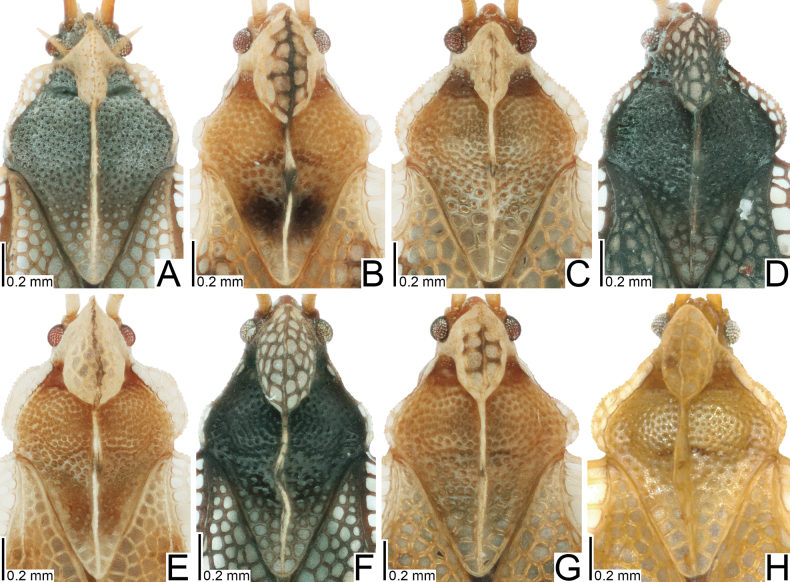
Female pronota of eight tingid species endemic to the Ogasawara Islands, Japan, dorsal view. A. *Acanthomoplax tomokunii*; B. *Omoplax desecta*; C. *O. hisasuei* sp. nov.; D. *O. inugusu* sp. nov.; E. *O. karubei*; F. *O. kobugashi* sp. nov.; G. *O. majorcarinae*; H. *O. mukojimensis*.

**Figure 5.**
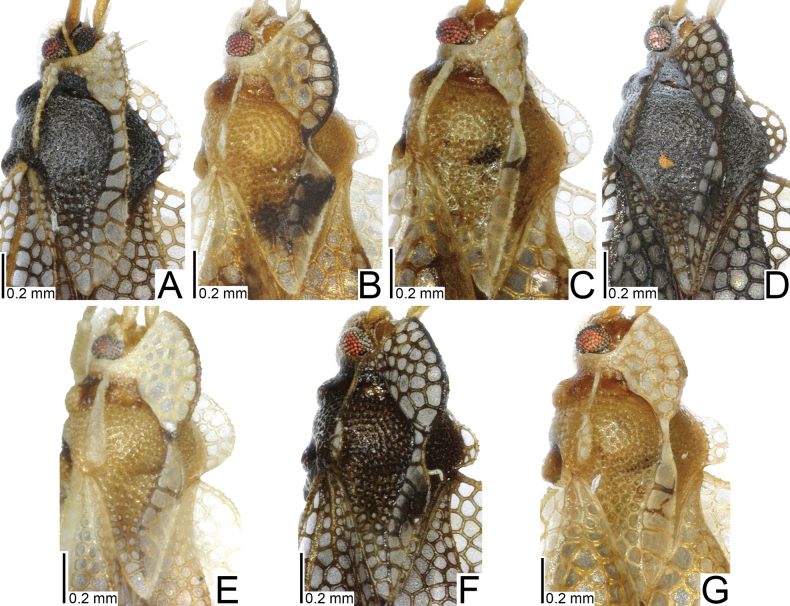
Male pronota of seven tingid species endemic to the Ogasawara Islands, Japan, dorsolateral view. A. *Acanthomoplax tomokunii* B. *Omoplax desecta* C. *O. hisasuei* sp. nov.; D. *O. inugusu* sp. nov.; E. *O. karubei* F. *O. kobugashi* sp. nov.; G. *O. majorcarinae*.

**Figure 6.**
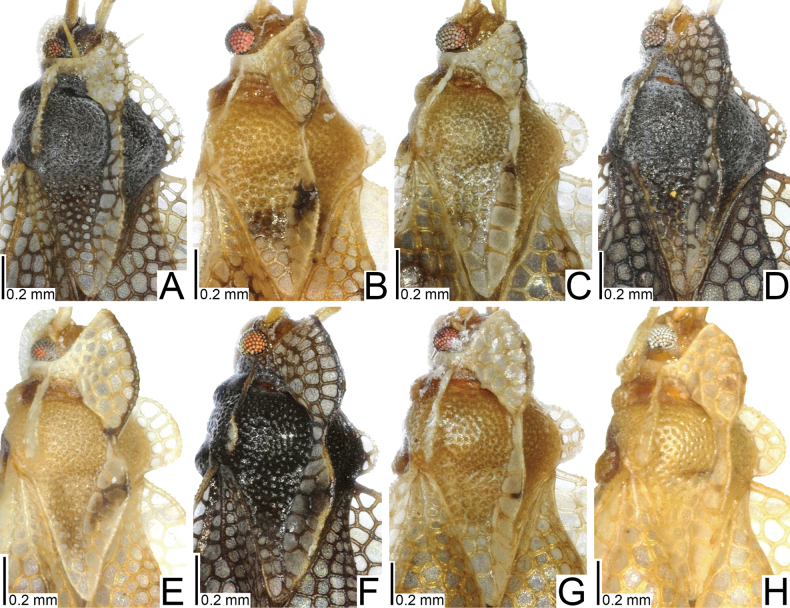
Female pronota of eight tingid species endemic to the Ogasawara Islands, Japan, dorsolateral view. A. *Acanthomoplax tomokunii*; B. *Omoplax desecta*; C. *O. hisasuei* sp. nov.; D. *O. inugusu* sp. nov.; E. *O. karubei*; F. *O. kobugashi* sp. nov.; G. *O. majorcarinae*; H. *O. mukojimensis*.

**Figure 7.**
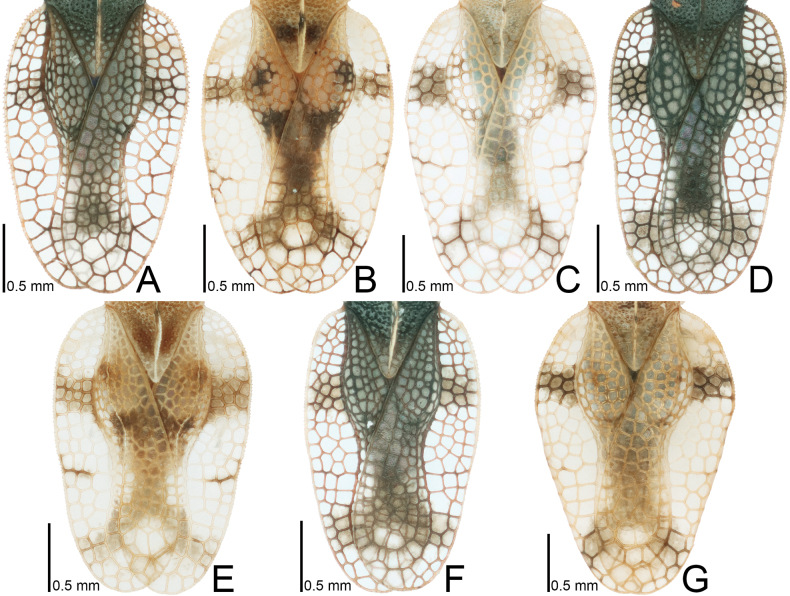
Male hemelytra of seven tingid species endemic to the Ogasawara Islands, Japan, dorsal view. A. *Acanthomoplax tomokunii*; B. *Omoplax desecta*; C. *O. hisasuei* sp. nov.; D. *O. inugusu* sp. nov.; E. *O. karubei*; F. *O. kobugashi* sp. nov.; G. *O. majorcarinae*.

**Figure 8.**
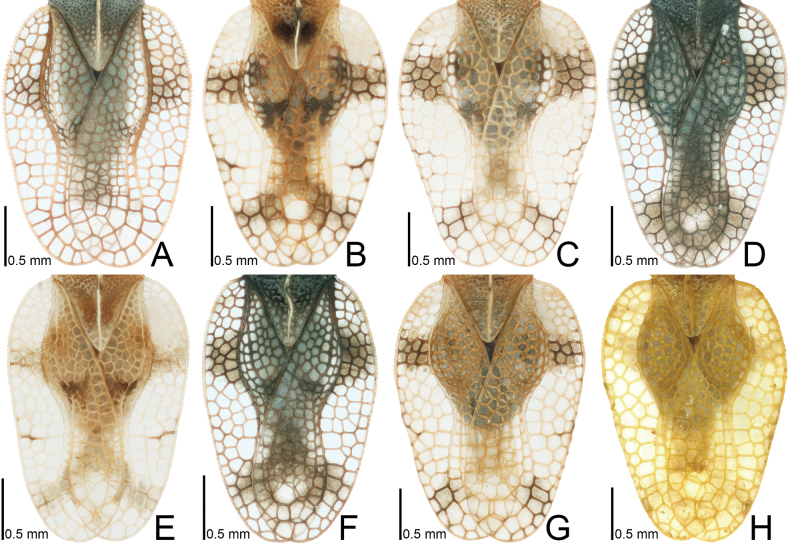
Female hemelytra of eight tingid species endemic to the Ogasawara Islands, Japan, dorsal view. A. *Acanthomoplax tomokunii*; B. *Omoplax desecta*; C. *O. hisasuei* sp. nov.; D. *O. inugusu* sp. nov.; E. *O. karubei*; F. *O. kobugashi* sp. nov.; G. *O. majorcarinae*; H. *O. mukojimensis*.

**Figure 9.**
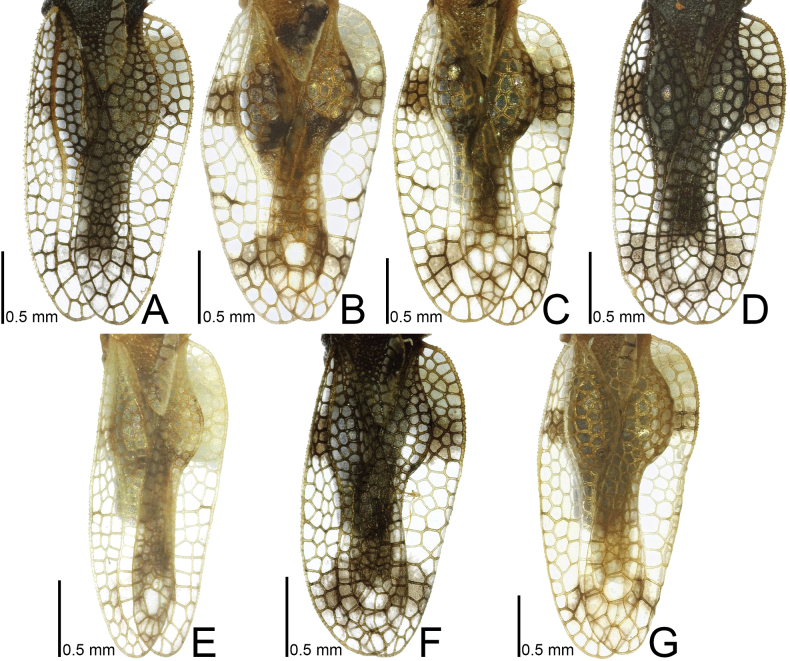
Male hemelytra of seven tingid species endemic to the Ogasawara Islands, Japan, dorsolateral view. A. *Acanthomoplax tomokunii*; B. *Omoplax desecta*; C. *O. hisasuei* sp. nov.; D. *O. inugusu* sp. nov.; E. *O. karubei*; F. *O. kobugashi* sp. nov.; G. *O. majorcarinae*.

**Figure 10.**
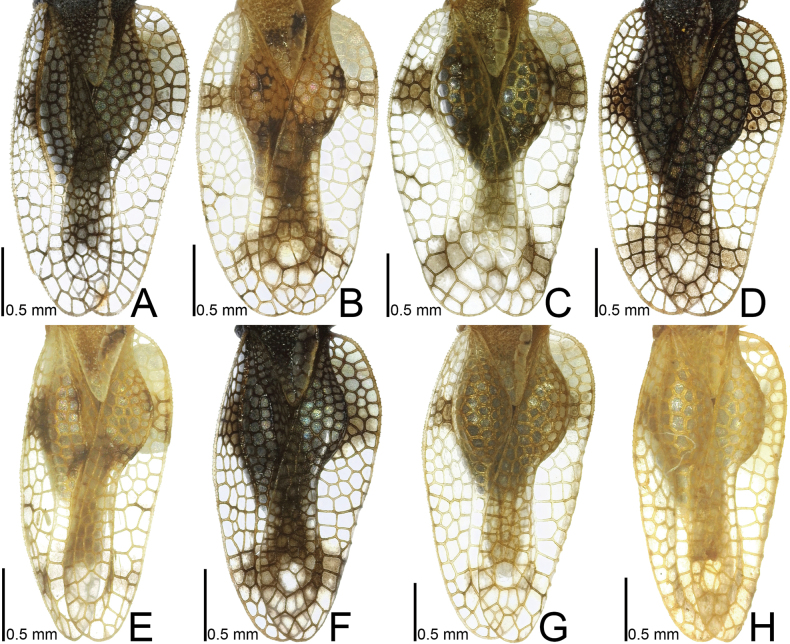
Female hemelytra of eight tingid species endemic to the Ogasawara Islands, Japan, dorsolateral view. A. *Acanthomoplax tomokunii*; B. *Omoplax desecta*; C. *O. hisasuei* sp. nov.; D. *O. inugusu* sp. nov.; E. *O. karubei*; F. *O. kobugashi* sp. nov.; G. *O. majorcarinae*; H. *O. mukojimensis*.

**Figure 11.**
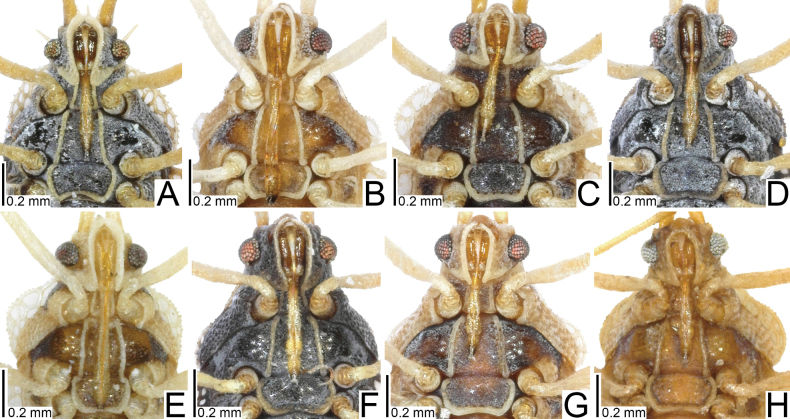
Rostra of eight tingid species endemic to the Ogasawara Islands, Japan, ventral view. A. *Acanthomoplax tomokunii*; B. *Omoplax desecta*; C. *O. hisasuei* sp. nov.; D. *O. inugusu* sp. nov.; E. *O. karubei*; F. *O. kobugashi* sp. nov.; G. *O. majorcarinae*; H. *O. mukojimensis*.

**Figure 12.**
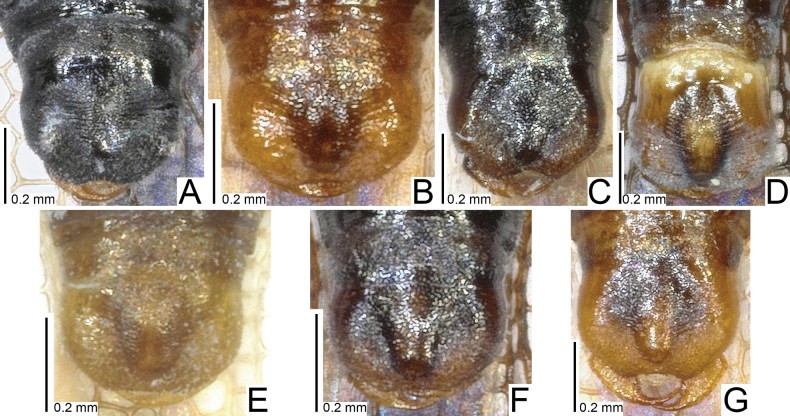
Male terminalia of seven tingid species endemic to the Ogasawara Islands, Japan, ventral view. A. *Acanthomoplax tomokunii*; B. *Omoplax desecta*; C. *O. hisasuei* sp. nov.; D. *O. inugusu* sp. nov.; E. *O. karubei*; F. *O. kobugashi* sp. nov.; G. *O. majorcarinae*.

**Figure 13.**
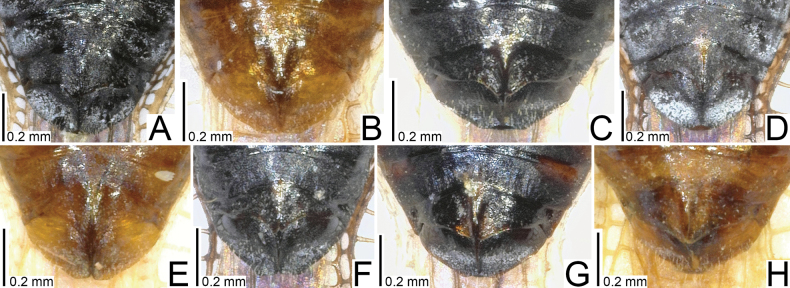
Female terminalia of eight tingid species endemic to the Ogasawara Islands, Japan, ventral view. A. *Acanthomoplax tomokunii*; B. *Omoplax desecta*; C. *O. hisasuei* sp. nov.; D. *O. inugusu* sp. nov.; E. *O. karubei*; F. *O. kobugashi* sp. nov.; G. *O. majorcarinae*; H. *O. mukojimensis*.

**Figure 14.**
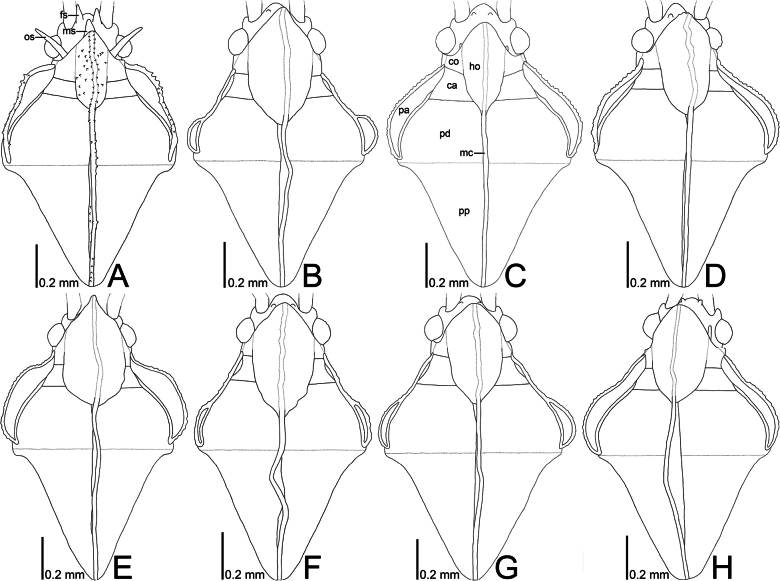
Line drawings of pronota of eight tingid species endemic to the Ogasawara Islands, Japan, dorsal view. A. *Acanthomoplax tomokunii*; B. *Omoplax desecta*; C. *O. hisasuei* sp. nov.; D. *O. inugusu* sp. nov.; E. *O. karubei*; F. *O. kobugashi* sp. nov.; G. *O. majorcarinae*; H. *O. mukojimensis*. Abbreviations: ca, calli; co, collar; fs, frontal spine; ho, hood; mc, median carina; ms, median spine; os, occipital spine; pa, paranotum; pd, pronotal disc; pp, posterior process.

*Omoplax desecta* is recognized among the other *Omoplax* species based on a combination of the following characteristics: rostrum reaching posterior margin of metasternum (Fig. 11B); pronotal disc pale brown (Figs 3B, 4B, 5B, 6B); hood more than 0.5 times as wide as maximum width of head across compound eyes, not reaching apex of clypeus (Fig. 14B); paranotum without areolae in middle part, with areolae in remaining parts; anterior margin of hemelytron weakly curved downward in apical half (Figs 7B, 8B, 9B, 10B); subcostal and discoidal areas of hemelytron not united; costal area narrower than combined width of subcostal and discoidal areas; Sc (subcosta) vein of hemelytron distinct in apical part of dorsal view; R+M (fused radius and media) vein of hemelytron indistinct, not carinate; and ventral surface of body in various shades of brown (Figs 12B, 13B).

#### Remarks.

In general appearance, *Omoplax desecta* is very similar to *O. majorcarinae*, whose distribution range is consistent with that of *O. desecta* in Chichijima Group (Guilbert 2001; Souma and Kamitani 2021; Souma 2022a); however, the former can be distinguished from the latter based on the following five main characters (Figs 3B, G, 4B, G, 5B, G, 6B, G, 7B, G, 8B, G, 9B, G, 10B, G, 11B, G, 14B, G): rostrum reaching posterior margin of metasternum (reaching middle part of mesosternum in *O. majorcarinae*); anterior margin of hemelytron weakly curved downward in apical half (strongly curved downward in apical half in *O. majorcarinae*); subcostal and discoidal areas of hemelytron not united (united in *O. majorcarinae*); Sc (subcosta) vein of hemelytron distinct in apical part of dorsal view (indistinct in apical part of dorsal view in *O. majorcarinae*); and R+M (fused radius and media) vein of hemelytron distinct, carinate (indistinct, not carinate in *O. majorcarinae*). Morphological differences between *O. desecta* and the five other *Omoplax* species are presented in the identification key below.

#### Distribution.

Japan: Ogasawara Islands: Chichijima Group (Anijima Island, Chichijima Island, Nishijima Island, Ototojima Island), Hahajima Group (Hahajima Island, Meijima Island, Mukohjima Island), Mukojima Group (Nakodojima Island) (Fig. 18) (Government of Japan 2010; Souma and Kamitani 2021; Souma 2022a; Shimamoto and Ishikawa 2023). *Omoplax desecta* is endemic to the Ogasawara Islands.

#### Host plant.

Rhaphiolepis indica var. tashiroi (Rosaceae) (Fig. 17C) and *Calophyllum inophyllum* (Clusiaceae) (Fig. 17D), also known as “Shimasharinbai” and “Terihaboku”, respectively, were confirmed as host plants for *Omoplax desecta* by the field and captive observations of adults and nymphs, suggesting the possibility of polyphagy for this lace bug species. However, no feeding behavior of *O. desecta* was observed on *Ardisia* sp. (Primulaceae), *Bischofia javanica* Blume (Phyllanthaceae), *Cinnamomum* sp. (Lauraceae), *Ligustrum* sp. (Oleaceae), and *Terminalia* sp. (Combretaceae), from which only a few adults were collected in previous studies (cf. Yasunaga et al. 1993; Guilbert 2001; Yamada and Tomokuni 2012). Therefore, these five tree species do not appear to be host plants for this lace bug species.

#### Bionomics.

*Omoplax desecta* inhabits an evergreen broad-leaved forest with a subtropical climate in the Ogasawara Islands (Souma and Kamitani 2021), and sucks sap on the abaxial side of the leaves of Rhaphiolepis indica var. tashiroi and *Calophyllum inophyllum*, causing irregular yellowing on the adaxial side (Fig. 17C, D). Adults were collected in all seasons (Drake 1956; Nishimura and Arai 1989; Yasunaga et al. 1993; Tomokuni 1994; Miyano 1998; Guilbert 2001; Souma and Kamitani 2021; Souma 2022a); nymphs were collected in March, April, and October. In Anijima Island, *O. desecta* has been confirmed in the stomach contents of the invasive green anole, *Anolis carolinensis* (Squamata, Dactyloidae) (Takahashi et al. 2014).

### ﻿ Omoplax hisasuei sp. nov.

Taxon classificationAnimaliaHemipteraTingidae

B56AA5BF-2090-5550-A354-519D2630BF7B

https://zoobank.org/F582D0F4-8C7B-47E5-B8A1-307D9B265ADA

Figs 1C, 2C, 3C, 4C, 5C, 6C, 7C, 8C, 9C, 10C, 11C, 12C, 13C, 14C, 15I

#### Type material.

***Holotype***, Japan • ♂; Ogasawara Isls., Hahajima Is., Mt. Kuwanoki; Neolitsea sericea var. aurata; 29 Sep. 2024; J. Souma leg.; SIHU. ***Paratypes***, Japan • 6 ♂♂ 5 ♀♀; same data as for holotype; SIHU • 1 ♂ 1 ♀; same locality and date data as for holotype; Y. Hisasue leg.; SIHU • 1 ♂; same locality, host plant, and collector data as for holotype; 8 Mar. 2025; SIHU • 2 ♂♂; same locality, host plant, and collector data as for holotype; 9 Mar. 2025; SIHU • 13 ♂♂ 14 ♀♀; same locality data as for holotype; 22 May 2025; S. Shimamoto leg.; SIHU.

#### Additional material examined.

***Non-types***, Japan • 1 fifth instar nymph; same locality and date data as for holotype; Y. Hisasue leg.; SIHU. The single nymph recorded above is in poor condition and is thus not described in the present study.

#### Diagnosis.

*Omoplax hisasuei* sp. nov. is recognized among the other *Omoplax* species based on a combination of the following characteristics: rostrum reaching middle part of mesosternum (Fig. 11C); pronotal disc pale brown (Figs 3C, 4C, 5C, 6C); hood less than 0.5 times as wide as maximum width of head across compound eyes, not reaching apex of clypeus (Fig. 14C); paranotum with areolae throughout its length; anterior margin of hemelytron strongly curved downward in apical half (Figs 7C, 8C, 9C, 10C); subcostal and discoidal areas of hemelytron united; costal area wider than fused subcostal and discoidal areas; Sc (subcosta) vein of hemelytron indistinct in apical part of dorsal view; R+M (fused radius and media) vein of hemelytron indistinct, not carinate; and ventral surface of body dark brown to black (Figs 12C, 13C).

#### Description.

**Male.** Head, antenna, calli, and legs in various shades of brown; pronotal disc, hood, median carina of pronotum, paranotum, posterior process, and hemelytron pale brown; markings on dorsum and ventral surface of body dark brown to black; compound eye dark red; areolae of pronotum and hemelytron transparent; pubescence on body yellowish (Figs 1C, 3C, 5C, 7C, 9C, 11C, 12C).

Body ovate; pubescence on body distinctly shorter than radius of compound eye (Figs 1C, 12C). Head (Figs 3C, 5C) glabrous; pair of frontal spines separated from each other at apices, not reaching apex of clypeus, occasionally reduced; median spine not reaching bases of frontal spines, occasionally reduced; pair of occipital spines not reaching anterior margin of compound eyes, occasionally reduced; antenniferous tubercle obtuse, curved inward, longer than frontal spines; vertex and clypeus smooth. Compound eye round in dorsal view. Antenna densely covered with minute pubescence on segments I to III and long pubescence on segment IV; pubescence on segment IV longer than pubescence on other parts of body; segment I cylindrical, shorter than segment IV; segment II cylindrical, shortest among antennal segments; segment III linear, longest amongst antennal segments; segment IV fusiform. Bucculae closed at anterior ends, with 3 rows of areolae at highest part. Rostrum (Fig. 11C) reaching middle part of mesosternum.

Pronotum (Figs 1C, 3C, 5C) glabrous. Pronotal disc coarsely punctate. Hood shorter than median carina of pronotum, higher than median carina, with 3–4 rows of areolae at highest part, less than 0.5 times as wide as maximum width of head across compound eyes, not reaching apex of clypeus, without robust denticles throughout its length; dorsal margin arched; posterior margin extending to anterior part of pronotal disc. Collar not covering compound eye. Median carina straight, extending to apex of posterior process, with 1–2 rows of areolae at highest part, without robust denticles throughout its length; dorsal margin arched. Calli smooth. Paranotum subvertical, widened posteriad, with a single row of areolae in anterior half and 2 rows in posterior half; outer margin gently curved outward throughout its length, without robust denticles throughout its length. Posterior process triangular; apex rounded.

Hemelytron (Figs 7C, 9C) glabrous, extending beyond apex of abdomen; anterior margin strongly curved downward in apical half; apices separated from each other at rest; subcostal and discoidal areas united; costal area wider than fused subcostal and discoidal areas, with 4–5 rows of areolae at widest part; fused subcostal and discoidal areas with 7 rows of areolae at widest part; sutural area with 4–5 rows of areolae at widest part; hypocostal lamina with a single row of areolae throughout its length; Sc (subcostal) and Hc (hypocosta) veins distinct throughout their respective length; R+M (fused radius and media) and Cu (cubitus) veins indistinct throughout their respective length, not carinate; Sc and R+M veins without robust denticles throughout their respective length; Sc vein indistinct in apical part of dorsal view.

Thoracic pleura smooth in anterior part, coarsely punctate in posterior part. Ostiolar peritreme oblong. Sternal laminae (Fig. 11C) lower than bucculae; pro- and mesosternal laminae open at both anterior and posterior ends; metasternal laminae as high as mesosternal laminae, open at anterior ends, fused with each other at posterior ends. Legs (Fig. 1C) smooth, covered with pubescence; femora thickest at middle. Abdomen ellipsoidal. Terminalia (Fig. 12C) pentagonal in ventral view, covered with pubescence.

Measurements (*n* = 24). Body length with hemelytra 3.05–3.30 mm; maximum width of body across hemelytra 1.65–1.75 mm; length of antennal segments I to IV 0.20 mm, 0.10 mm, 1.20 mm, and 0.70 mm, respectively; pronotal length 1.10–1.20 mm; pronotal width across paranota 0.80–0.85 mm; hemelytral length 2.40–2.55 mm; maximum width of hemelytron 0.95–1.00 mm.

**Female.** General habitus very similar to that of male (Figs 2C, 4C, 6C, 8C, 10C, 13C) except for the following characters: body wider than in male; antennal segments III and IV shorter than in male; hemelytron usually wider than in male; and apical part of abdomen pentagonal in ventral view.

Measurements (*n* = 20). Body length with hemelytra 3.15–3.35 mm; maximum width of body across hemelytra 1.85–1.95 mm; length of antennal segments I to IV 0.20 mm, 0.10 mm, 1.10 mm, and 0.60 mm, respectively; pronotal length 1.15–1.20 mm; pronotal width across paranota 0.80–0.85 mm; hemelytral length 2.45–2.55 mm; maximum width of hemelytron 1.00–1.05 mm.

#### Remarks.

Among all the species of *Omoplax*, *O. hisasuei* sp. nov. strongly resembles *O. majorcarinae* and *O. mukojimensis* in terms of its general habitus. However, based on a comparison of the type material of the new species together with the holotype or non-type material and the original descriptions (Guilbert 2001; Souma 2022a) of *O. majorcarinae* and *O. mukojimensis*, three main characteristics were recognized to easily differentiate *O. hisasuei* sp. nov. from *O. majorcarinae* and *O. mukojimensis* (Figs 3C, G, 4C, G, H, 5C, G, 6C, G, H, 7C, G, 8C, G, H, 9C, G, 10C, G, H, 14C, G, H): hood less than 0.5 times as wide as maximum width of head across compound eyes (more than 0.5 times as wide as maximum width of head across compound eyes in *O. majorcarinae* and *O. mukojimensis*); paranotum with areolae throughout its length (without areolae in middle part, with areolae in remaining parts in *O. majorcarinae*); and costal area wider than fused subcostal and discoidal areas (narrower than fused subcostal and discoidal areas in *O. majorcarinae* and *O. mukojimensis*). Morphological differences between *O. hisasuei* sp. nov. and the four other *Omoplax* species are presented in the identification key below.

#### Distribution.

Japan: Ogasawara Islands: Hahajima Group (Hahajima Island) (Fig. 19). *Omoplax hisasuei* sp. nov. is endemic to Hahajima Island.

#### Etymology.

This new species is named in honor of Yu Hisasue, a Japanese hymenopterist who collected some of the paratypes and has contributed to clarifying the species diversity of insects from the Ogasawara Islands.

#### Host plant.

Only Neolitsea sericea var. aurata (Lauraceae) (Fig. 17E), which is also known as “Kinshokudamo”, was confirmed as a host plant for *Omoplax hisasuei* sp. nov. through field and captive observations of adults and nymphs, suggesting the possibility of monophagy for this lace bug species.

#### Bionomics.

*Omoplax hisasuei* sp. nov. inhabits an evergreen broad-leaved forest with a subtropical climate in the Ogasawara Islands and sucks sap on the abaxial side of the leaves of Neolitsea sericea var. aurata, causing irregular yellowing on the adaxial side (Fig. 17E). Adults were collected in March, May, and September; a single nymph was collected in September.

### ﻿ Omoplax inugusu sp. nov.

Taxon classificationAnimaliaHemipteraTingidae

EB0E928C-9918-5EA5-A121-E0509E4D7ECD

https://zoobank.org/12386280-77CA-4F43-9167-5B0CC509FFDC

Figs 1D, 2D, 3D, 4D, 5D, 6D, 7D, 8D, 9D, 10D, 11D, 12D, 13D, 14D, 16A, B

Omoplax majorcarinae Guilbert, 2001: Souma and Kamitani (2021: 9) (distribution: part); Souma (2022a: 126) (distribution: part); Shimamoto and Ishikawa (2023: 94) (catalog: part); Souma (2023: 9) (monograph). Misidentifications.

#### Type material.

***Holotype***, Japan • ♂; Ogasawara Isls., Hahajima Is., Tamagawa Dam; *Machilus boninensis*; 1 Oct. 2024; J. Souma leg.; SIHU. ***Paratypes***, Japan • 2 ♂♂ 4 ♀♀; Ogasawara Isls., Hahajima Is., Kitamura; 4 Jun. 1976; T. Nakane leg.; 1 ♂ 3 ♀♀ referring to Souma (2022a); NSMT • 1 ♂ 5 ♀♀; Ogasawara Isls., Hahajima Is., Mt. Chibusa; 21 Jun. 1994; Y. Kaneko leg.; referring to Souma and Kamitani (2021); TUA • 2 ♀♀; same locality data as for preceding; 7 Jul. 1997; K. Matsumoto leg.; referring to Souma (2022a); NSMT • 1 ♂ 1 ♀; same locality and collector data as for preceding; 18 Jun. 2001; referring to Souma and Kamitani (2021); TUA • 1 ♂; same locality data as for preceding; 12 Jun. 2024; Y. Hisasue leg.; SIHU • 3 ♀♀; same locality data as for preceding; *Machilus boninensis*; 2 Oct. 2024; J. Souma leg.; SIHU • 1 ♀; Ogasawara Isls., Hahajima Is., “石門” [= Sekimon]; 27 Jun. 2009; Japan Forest Technology Association leg.; referring to Souma (2022a); KPMNH • 2 ♂♂ 2 ♀♀; Ogasawara Isls., Hahajima Is., Mt. Sekimon; alt. 356 m; 22 Jun. 2022; S. Tomura leg.; SIHU • 2 ♀♀; same data as for holotype; SIHU • 1 ♂ 3 ♀♀; same locality data as for holotype; 27 Jun. 1996; K. Morimoto leg.; referring to Souma and Kamitani (2021); ELKU • 2 ♂♂ 4 ♀♀; same locality data as for holotype; 14 Jul. 2024; Y. Uehara leg.; SIHU • 1 ♂; Ogasawara Isls., Hahajima Is., Mt. Kichibe; 16 Jun. 2024; N. Tsuji leg.; SIHU • 1 ♂ 1 ♀; Ogasawara Isls, Hahajima Is., Nishidai; 10 Jun. 2025; N. Tsuji leg.; SIHU.

#### Diagnosis.

*Omoplax inugusu* sp. nov. is recognized among the other *Omoplax* species based on a combination of the following characteristics: rostrum reaching middle part of mesosternum (Fig. 11D); pronotal disc black (Figs 3D, 4D, 5D, 6D); hood more than 0.5 times as wide as maximum width of head across compound eyes, not reaching apex of clypeus (Fig. 14D); paranotum with areolae throughout its length; anterior margin of hemelytron not curved downward in apical half (Figs 7D, 8D, 9D, 10D); subcostal and discoidal areas of hemelytron not united; costal area narrower than combined width of subcostal and discoidal areas; Sc (subcosta) vein of hemelytron visible in apical part of dorsal view; R+M (fused radius and media) vein of hemelytron indistinct, not carinate; and ventral surface of body dark brown to black (Figs 12D, 13D).

#### Description.

**Male.** Markings on dorsum, head, hood, median carina of pronotum, paranotum, calli, posterior process, hemelytron, and ventral surface of body dark brown to black; antenna and legs in various shades of brown; pronotal disc black; compound eye dark red; areolae of pronotum and hemelytron transparent; pubescence on body yellowish (Figs 1D, 3D, 5D, 7D, 9D, 11D, 12D).

Body ovate; pubescence on body shorter than radius of compound eye (Figs 1D, 12D). Head (Figs 3D, 5D) glabrous; pair of frontal spines separated from each other at apices, not reaching apex of clypeus, occasionally reduced; median spine not reaching bases of frontal spines, occasionally reduced; pair of occipital spines not reaching anterior margin of compound eyes, occasionally reduced; antenniferous tubercle obtuse, curved inward, longer than frontal spines; vertex and clypeus smooth. Compound eye round in dorsal view. Antenna densely covered with minute pubescence on segments I to III and long pubescence on segment IV; pubescence on segment IV longer than pubescence on other parts of body; segment I cylindrical, shorter than segment IV; segment II cylindrical, shortest among antennal segments; segment III linear, longest amongst antennal segments; segment IV fusiform. Bucculae closed at anterior ends, with 3 rows of areolae at highest part. Rostrum (Fig. 11D) reaching middle part of mesosternum.

Pronotum (Figs 1D, 3D, 5D) glabrous. Pronotal disc coarsely punctate. Hood shorter than median carina of pronotum, higher than median carina, with 4–5 rows of areolae at highest part, more than 0.5 times as wide as maximum width of head across compound eyes, not reaching apex of clypeus, without robust denticles throughout its length; dorsal margin arched; posterior margin extending to anterior part of pronotal disc. Collar not covering compound eye. Median carina straight, extending to apex of posterior process, with 2 rows of areolae at highest part, without robust denticles throughout its length; dorsal margin arched. Calli smooth. Paranotum subvertical, widened posteriad, with a single row of areolae in anterior half and 2–3 rows in posterior half; outer margin gently curved outward throughout its length, without robust denticles throughout its length. Posterior process triangular; apex rounded.

Hemelytron (Figs 7D, 9D) glabrous, extending beyond apex of abdomen; anterior margin not curved downward in apical half; apices close to each other at rest; subcostal and discoidal areas not united; costal area narrower than combined width of subcostal and discoidal areas, with 4–5 rows of areolae at widest part; subcostal area with 3 rows of areolae at widest part; discoidal area with 4 rows of areolae at widest part; sutural area with 5 rows of areolae at widest part; hypocostal lamina with a single row of areolae throughout its length; Sc (subcostal) and Hc (hypocosta) veins distinct throughout their respective length; R+M (fused radius and media) and Cu (cubitus) veins indistinct throughout their respective length, not carinate; Sc and R+M veins without robust denticles throughout their respective length; Sc vein distinct in apical part of dorsal view.

Thoracic pleura smooth in anterior part, coarsely punctate in posterior part. Ostiolar peritreme oblong. Sternal laminae (Fig. 11D) lower than bucculae; pro- and mesosternal laminae open at both anterior and posterior ends; metasternal laminae as high as mesosternal laminae, open at anterior ends, fused with each other at posterior ends. Legs (Fig. 1D) smooth, covered with pubescence; femora thickest at middle. Abdomen ellipsoidal. Terminalia (Fig. 12D) pentagonal in ventral view, covered with pubescence.

Measurements (*n* = 14). Body length with hemelytra 2.75–3.10 mm; maximum width of body across hemelytra 1.30–1.55 mm; length of antennal segments I to IV 0.20 mm, 0.10 mm, 1.20 mm, and 0.60 mm, respectively; pronotal length 1.20–1.25 mm; pronotal width across paranota 0.75–0.80 mm; hemelytral length 2.05–2.30 mm; maximum width of hemelytron 0.80–0.90 mm.

**Female.** General habitus very similar to that of male (Figs 2D, 4D, 6D, 8D, 10D, 13D) except for the following characters: body usually longer and wider than in male; antennal segments III and IV shorter than in male; hemelytron usually longer and wider than in male; and apical part of abdomen pentagonal in ventral view.

Measurements (*n* = 31). Body length with hemelytra 2.90–3.15 mm; maximum width of body across hemelytra 1.55–1.65 mm; length of antennal segments I to IV 0.20 mm, 0.10 mm, 1.10 mm, and 0.50 mm, respectively; pronotal length 1.20–1.30 mm; pronotal width across paranota 0.80–0.85 mm; hemelytral length 2.25–2.45 mm; maximum width of hemelytron 0.85–0.95 mm.

#### Remarks.

In the previous studies (Souma and Kamitani 2021; Souma 2022a), *Omoplax inugusu* sp. nov. was identified as *O. majorcarinae*, but the former differs from the original description and the illustrations of the latter (Guilbert 2001) based on the following characters: body length with hemelytra 2.75–3.15 mm (3.45 mm in type material) (Figs 1D, 2D); maximum width of body across hemelytra 1.30–1.65 mm (1.85 mm in type material); pronotal disc black (Figs 3D, 4D, 5D, 6D); paranotum with areolae throughout its length (Fig. 14D); anterior margin of hemelytron not curved downward in apical half (Figs 7D, 8D, 9D, 10D); and Sc (subcosta) vein of hemelytron distinct in apical part of dorsal view. These features were considered intraspecific variations of *O. majorcarinae* in the previous studies (cf. Souma and Kamitani 2021; Souma 2022a), but constitute interspecific variation in the present study based on the examination of dozens of specimens which show two morphological species, each respectively collected from different plant species.

Among all the *Omoplax* species, *O. inugusu* sp. nov. strongly resembles *O. kobugashi* sp. nov. in terms of its general habitus. However, based on a comparison of the type materials of *O. inugusu* sp. nov. and *O. kobugashi* sp. nov., three main characteristics were recognized to easily differentiate *O. inugusu* sp. nov. from *O. kobugashi* sp. nov. (Figs 3D, F, 4D, F, 5D, F, 6D, F, 7D, F, 8D, F, 9D, F, 10D, F, 11D, F, 14D, F): rostrum reaching middle part of mesosternum (reaching posterior margin of mesosternum in *O. kobugashi* sp. nov.); paranotum with areolae throughout its length (without areolae in middle part, with areolae in remaining parts in *O. kobugashi* sp. nov.); and anterior margin of hemelytron not curved downward in apical half (weakly curved downward in apical half in *O. kobugashi* sp. nov.). Morphological differences between *O. inugusu* sp. nov. and the five other *Omoplax* species are presented in the identification key below.

#### Distribution.

Japan: Ogasawara Islands: Hahajima Group (Hahajima Island) (Fig. 19) (Souma and Kamitani 2021; Souma 2022a). *Omoplax inugusu* sp. nov. is endemic to Hahajima Island.

#### Etymology.

The specific epithet is the Japanese plant name “Munin-inugusu” [= *Machilus boninensis*], referring to the host plant of the new species; a noun in apposition.

#### Host plant.

Only *Machilus boninensis* (Lauraceae) (Fig. 17F), which is also known as “Munin-inugusu”, was confirmed as a host plant for *Omoplax inugusu* sp. nov. by the field and captive observations of adults, suggesting the possibility of monophagy for this lace bug species.

#### Bionomics.

*Omoplax inugusu* sp. nov. inhabits an evergreen broad-leaved forest with a subtropical climate in the Ogasawara Islands (Souma and Kamitani 2021), and sucks sap on the abaxial side of the leaves of *Machilus boninensis*, causing irregular yellowing on the adaxial side (Fig. 17F). Adults were collected in June, July, and October (Souma and Kamitani 2021; Souma 2022a); nymphs are unknown.

### ﻿ Omoplax karubei

Taxon classificationAnimaliaHemipteraTingidae

Souma, 2022

38E875D8-3E1B-5BF2-A11A-862D26ED8A87

Figs 1E, 2E, 3E, 4E, 5E, 6E, 7E, 8E, 9E, 10E, 11E, 12E, 13E, 14E

Omoplax karubei Souma, 2022a: 118. Holotype: ♂; type locality: Japan • “東京都小笠原村, 聟島” [= Ogasawara Islands, Mukojima Group, Mukojima Island]; KPMNH.

#### References.

Shimamoto and Ishikawa (2023: 94) (catalog); Souma (2023: 9) (monograph).

#### Material examined.

***Non-types***, Japan • 2 ♂♂ 2 ♀♀; Ogasawara Isls, Mukojima Is.; Rhaphiolepis indica var. tashiroi; 15–16 Jul. 2024; S. Yagi, M. Kimura & J.-H. Park leg.; SIHU.

#### Diagnosis.

*Omoplax karubei* is recognized among the other *Omoplax* species based on a combination of the following characteristics: rostrum reaching posterior margin of metasternum (Fig. 11E); pronotal disc pale brown (Figs 3E, 4E, 5E, 6E); hood more than 0.5 times as wide as maximum width of head across compound eyes, reaching beyond apex of clypeus (Fig. 14E); paranotum with areolae throughout its length; anterior margin of hemelytron not curved downward in apical half (Figs 7E, 8E, 9E, 10E); subcostal and discoidal areas of hemelytron not united; costal area narrower than combined width of subcostal and discoidal areas; Sc (subcosta) vein of hemelytron distinct in apical part of dorsal view; R+M (fused radius and media) vein of hemelytron distinct, carinate; and ventral surface of body in various shades of brown (Figs 12E, 13E).

#### Remarks.

*Omoplax karubei* can be distinguished from *O. mukojimensis*, whose distribution range is consistent with that of *O. karubei* (Souma 2022a), based on the following six main characters (Figs 3E, 4E, H, 5E, 6E, H, 7E, 8E, H, 9E, 10E, H, 11E, H, 14E, H): rostrum reaching posterior margin of metasternum (reaching middle part of mesosternum in *O. mukojimensis*); hood reaching beyond apex of clypeus (not reaching apex of clypeus in *O. mukojimensis*); anterior margin of hemelytron not curved downward in apical half (strongly curved downward in apical half in *O. mukojimensis*); subcostal and discoidal areas of hemelytron not united (united in *O. mukojimensis*); Sc (subcosta) vein of hemelytron distinct in apical part of dorsal view (indistinct in apical part of dorsal view in *O. mukojimensis*); and R+M (fused radius and media) vein of hemelytron distinct, carinate (indistinct, not carinate in *O. mukojimensis*). Morphological differences between *O. karubei* and the five other *Omoplax* species are presented in the identification key below.

**Figure 15.**
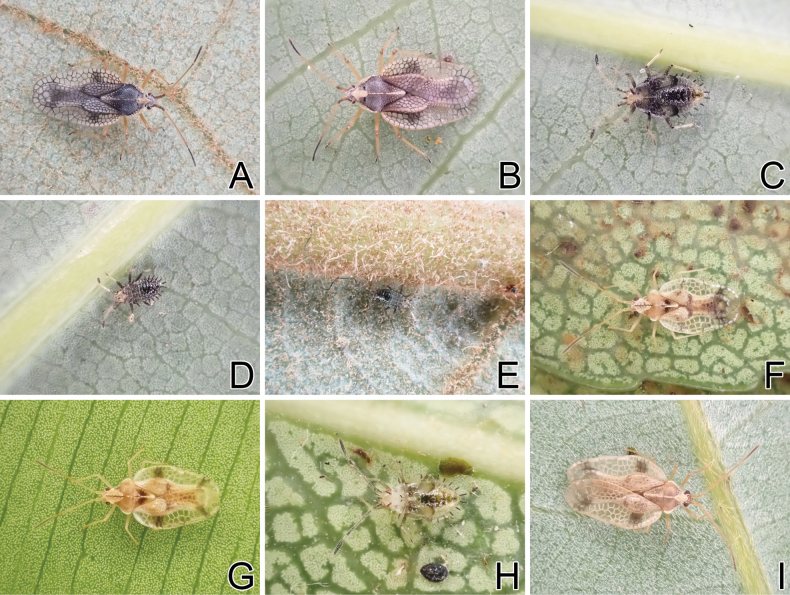
Living individuals of three tingid species endemic to the Ogasawara Islands, Japan. A. *Acanthomoplax tomokunii*, male; B. *A. tomokunii*, female; C. *A. tomokunii*, fifth instar nymph; D. *A. tomokunii*, third instar nymph; E. *A. tomokunii*, second instar nymph; F. *Omoplax desecta*, male; G. *O. desecta*, female; H. *O. desecta*, fifth instar nymph; I. *O. hisasuei* sp. nov., male.

#### Distribution.

Japan: Ogasawara Islands: Mukojima Group (Mukojima Island) (Fig. 19) (Souma 2022a). *Omoplax karubei* is endemic to Mukojima Island.

#### Host plant.

Only Rhaphiolepis indica var. tashiroi (Rosaceae) (Fig. 17G), which is also known as “Shimasharinbai”, was confirmed as a host plant for *Omoplax karubei* by the field observation of adults, suggesting the possibility of monophagy for this lace bug species.

#### Bionomics.

*Omoplax karubei* inhabits an evergreen broad-leaved forest with a subtropical climate in the Ogasawara Islands (Souma 2022a), and sucks sap on the abaxial side of the leaves of Rhaphiolepis indica var. tashiroi, causing irregular yellowing on the adaxial side (Fig. 17G). Adults were collected in June and July; nymphs are unknown (Souma 2022a).

### ﻿ Omoplax kobugashi sp. nov.

Taxon classificationAnimaliaHemipteraTingidae

718C9CE1-C7BF-5E0F-A66F-9390989F29BE

https://zoobank.org/E47CCFD8-B412-4A3A-81C3-EB1532091F69

Figs 1F, 2F, 3F, 4F, 5F, 6F, 7F, 8F, 9F, 10F, 11F, 12F, 13F, 14F, 16C–F

Omoplax majorcarinae Guilbert, 2001: Souma and Kamitani (2021: 9) (distribution: part); Souma (2022a: 126) (distribution: part); Shimamoto and Ishikawa (2023: 93) (catalog: part); Souma (2023: 9) (monograph). Misidentifications.

#### Type material.

***Holotype***, Japan • ♂; Ogasawara Isls., Chichijima Is., Mt. Mikazuki; *Machilus kobu*; 22 Sep. 2024; J. Souma leg.; SIHU. ***Paratypes***, Japan • 1 ♂; Ogasawara Isls., Chichijima Is.; 4 May 1976; Y. Hori leg.; referring to Souma (2022a); NSMT • 1 ♀; Ogasawara Isls., Chichijima Is., “大村” [= Omura]; 18 Jun. 1976; Y. Kurosawa leg.; referring to Souma (2022a); NSMT • 1 ♂ 1 ♀; Ogasawara Isls., Chichijima Is., Tokoyo Falls–Tastumizaki; 29 Jun. 1994; Y. Kaneko leg.; referring to Souma and Kamitani (2021); TUA • 1 ♀; Ogasawara Isls., Chichijima Is., Mt. Chuosan; 30 Jun. 1994; Y. Kaneko leg.; referring to Souma and Kamitani (2021); TUA • 1 ♂ 4 ♀♀; same locality data as for preceding; 27 Jun. 2001; K. Matsumoto leg.; 1 ♂ 2 ♀♀ referring to Souma and Kamitani (2021); TUA • 1 ♀; same locality data as for preceding; 22 Jun. 2024; Y. Hisasue leg.; SIHU • 1 ♀; Ogasawara Isls., Chichijima Is., Mt. Tsutsuji; 28 Jul. 1996; T. Kishimoto leg.; referring to Souma (2022a); NSMT • 1 ♂ 1 ♀; Ogasawara Isls., Chichijima Is., Mt. Yoake; 14 Jun. 1999; K. Morimoto leg.; referring to Souma and Kamitani (2021); ELKU • 1 ♀; Ogasawara Isls., Anijima Is., “ヤギ柵手前” [= Front of goat fence located at Central plateau]; 8 Jul. 2009; Japan Forest Technology Association leg.; referring to Souma (2022a); KPMNH • 1 ♂; Ogasawara Isls., Anijima Is., Mt. Mikaeri, Anbu; Malaise trap; 4 Jul. 2014; D. Watabiki leg.; referring to Souma and Kamitani (2021); TUA • 1 ♀; same locality, trap, and collector data as for preceding; 28 Jul. 2014; referring to Souma and Kamitani (2021); TUA • 1 ♀; Ogasawara Isls., Anijima Is., Mt. Omaru; 25 Jun. 2023; Y. Hisasue leg.; SIHU • 1 ♂; Ogasawara Isls., Anijima Is., Mt. Maruyama; 2 Jul. 2024; Y. Hisasue leg.; SIHU • 1 ♂ 1 ♀; Ogasawara Isls., Anijima Is., Southwest foot of Mt. Token; 8 Jul. 2024; N. Tsuji leg.; SIHU • 1 ♂; Ogasawara Isls., Chichijima Is., Mt. Tsuitate; 19 Jul. 2024; Y. Hisasue leg.; SIHU • 1 ♀; same data as for holotype; SIHU • 3 ♂♂ 4 ♀♀; Ogasawara Isls., Anijima Is., Tamana Beach–Mt. Mikaeri; *Machilus kobu*; 24 Sep. 2024; J. Souma leg.; SIHU.

#### Additional material examined.

***Non-types***, Japan • 1 fifth instar nymph 1 fourth instar nymph; Ogasawara Isls., Anijima Is., Tamana Beach–Mt. Mikaeri; *Machilus kobu*; 24 Sep. 2024; J. Souma leg.; SIHU. The two nymphs recorded above are in poor condition and are thus not described in the present study.

#### Diagnosis.

*Omoplax kobugashi* sp. nov. is recognized among the other *Omoplax* species based on a combination of the following characteristics: rostrum reaching posterior margin of mesosternum (Fig. 11F); pronotal disc black (Figs 3F, 4F, 5F, 6F); hood more than 0.5 times as wide as maximum width of head across compound eyes, not reaching apex of clypeus (Fig. 14F); paranotum without areolae in middle part, with areolae in remaining parts; anterior margin of hemelytron weakly curved downward in apical half (Figs 7F, 8F, 9F, 10F); subcostal and discoidal areas of hemelytron not united; costal area narrower than combined width of subcostal and discoidal areas; Sc (subcosta) vein of hemelytron distinct in apical part of dorsal view; R+M (fused radius and media) vein of hemelytron indistinct, not carinate; and ventral surface of body dark brown to black (Figs 12F, 13F).

**Figure 16.**
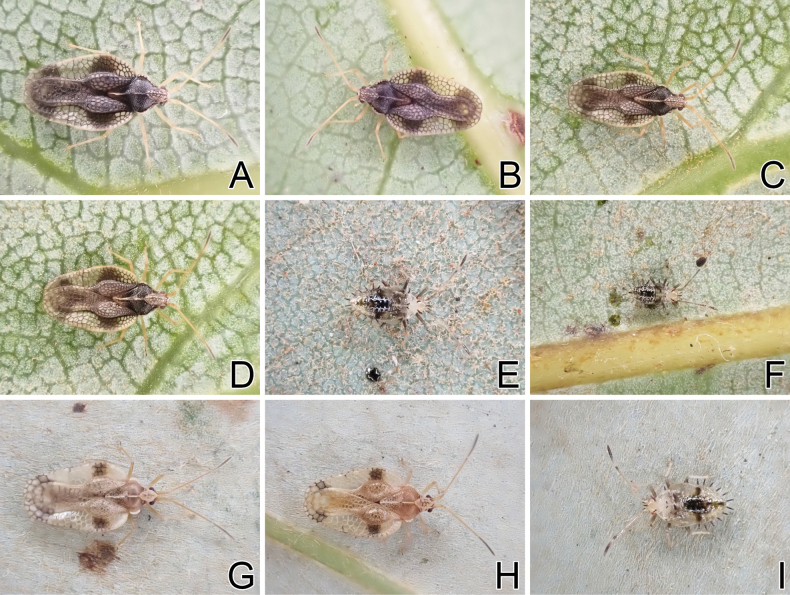
Living individuals of three tingid species endemic to the Ogasawara Islands, Japan. A. *Omoplax inugusu* sp. nov., male; B. *O. inugusu* sp. nov., female; C. *O. kobugashi* sp. nov., male; D. *O. kobugashi* sp. nov., female; E. *O. kobugashi* sp. nov., fifth instar nymph; F. *O. kobugashi* sp. nov., fourth instar nymph; G. *O. majorcarinae*, male; H. *O. majorcarinae*, female; I. *O. majorcarinae*, fifth instar nymph.

#### Description.

**Male.** Markings on dorsum, head, hood, median carina of pronotum, paranotum, calli, posterior process, hemelytron, and ventral surface of body dark brown to black; antenna and legs in various shades of brown; pronotal disc black; compound eye dark red; areolae of pronotum and hemelytron transparent; pubescence on body yellowish (Figs 1F, 3F, 5F, 7F, 9F, 11F, 12F).

**Figure 17.**
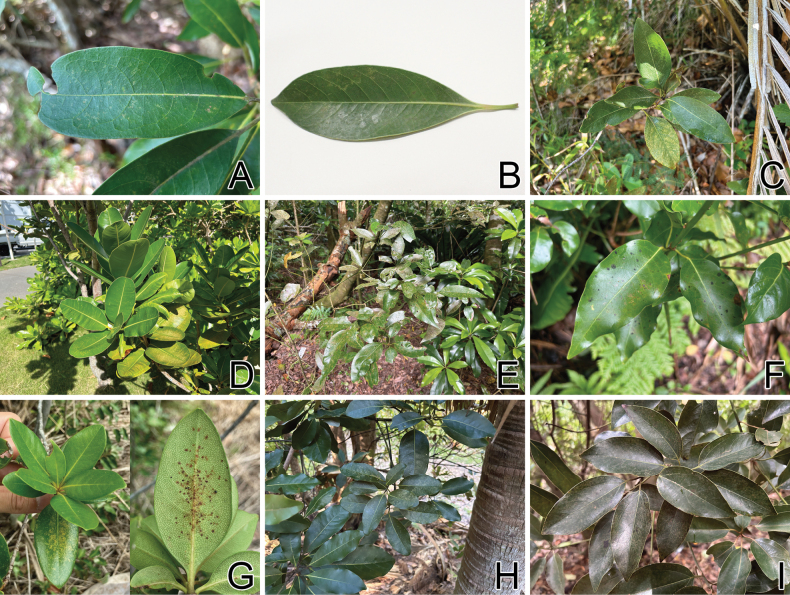
Host plants of seven tingid species endemic to the Ogasawara Islands, Japan. A. *Machilus kobu* from Ototojima Island, damaged by *Acanthomoplax tomokunii*; B. *M. thunbergii* planted in northern Honshu, damaged by *A. tomokunii* in captivity; C. Rhaphiolepis indica var. tashiroi from Chichijima Island, damaged by *Omoplax desecta*; D. *Calophyllum inophyllum* from Chichijima Island, damaged by *O. desecta*; E. Neolitsea sericea var. aurata Hahajima Island, damaged by *O. hisasuei* sp. nov.; F. *M. boninensis* from Hahajima Island, damaged by *O. inugusu* sp. nov.; G. R. indica var. tashiroi from Mukojima Island, damaged by *O. karubei* (photographs taken by Jinhyeong Park); H. *M. kobu* from Chichijima Island, damaged by *O. kobugashi* sp. nov.; I. N. sericea var. aurata Ototojima Island, damaged by *O. majorcarinae*.

Body ovate; pubescence on body shorter than radius of compound eye (Figs 1F, 12F). Head (Figs 3F, 5F) glabrous; pair of frontal spines separated from each other at apices, not reaching apex of clypeus, occasionally reduced; median spine not reaching bases of frontal spines, occasionally reduced; pair of occipital spines not reaching anterior margin of compound eyes, occasionally reduced; antenniferous tubercle obtuse, curved inward, longer than frontal spines; vertex and clypeus smooth. Compound eye round in dorsal view. Antenna densely covered with minute pubescence on segments I to III and long pubescence on segment IV; pubescence on segment IV longer than pubescence on other parts of body; segment I cylindrical, shorter than segment IV; segment II cylindrical, shortest among antennal segments; segment III linear, longest amongst antennal segments; segment IV fusiform. Bucculae closed at anterior ends, with 3 rows of areolae at highest part. Rostrum (Fig. 11F) reaching posterior margin of mesosternum.

Pronotum (Figs 1F, 3F, 5F) glabrous. Pronotal disc coarsely punctate. Hood shorter than median carina of pronotum, higher than median carina, with 4–5 rows of areolae at highest part, more than 0.5 times as wide as maximum width of head across compound eyes, not reaching apex of clypeus, without robust denticles throughout its length; dorsal margin arched; posterior margin extending to anterior part of pronotal disc. Collar not covering compound eye. Median carina straight, extending to apex of posterior process, with 2 rows of areolae at highest part, without robust denticles throughout its length; dorsal margin arched. Calli smooth. Paranotum subvertical, widened posteriad, with a single row of areolae in anterior part and 1–2 rows in posterior part, without areolae in middle part; outer margin gently curved outward in posterior part and straight in remaining parts, without robust denticles throughout its length. Posterior process triangular; apex rounded.

Hemelytron (Figs 7F, 9F) glabrous, extending beyond apex of abdomen; anterior margin weakly curved downward in apical half; apices close to each other at rest; subcostal and discoidal areas not united; costal area narrower than combined width of subcostal and discoidal areas, with 4–5 rows of areolae at widest part; subcostal area with 3 rows of areolae at widest part; discoidal area with 4 rows of areolae at widest part; sutural area with 5 rows of areolae at widest part; hypocostal lamina with a single row of areolae throughout its length; Sc (subcostal) and Hc (hypocosta) veins distinct throughout their respective length; R+M (fused radius and media) and Cu (cubitus) veins indistinct throughout their respective length, not carinate; Sc and R+M veins without robust denticles throughout their respective length; Sc vein distinct in apical part of dorsal view.

Thoracic pleura smooth in anterior part, coarsely punctate in posterior part. Ostiolar peritreme oblong. Sternal laminae (Fig. 11F) lower than bucculae; pro- and mesosternal laminae open at both anterior and posterior ends; metasternal laminae as high as mesosternal laminae, open at anterior ends, fused with each other at posterior ends. Legs (Fig. 1F) smooth, covered with pubescence; femora thickest at middle. Abdomen ellipsoidal. Terminalia (Fig. 12F) pentagonal in ventral view, covered with pubescence.

Measurements (*n* = 13). Body length with hemelytra 2.60–2.80 mm; maximum width of body across hemelytra 1.20–1.35 mm; length of antennal segments I to IV 0.20 mm, 0.10 mm, 1.10 mm, and 0.60 mm, respectively; pronotal length 1.15–1.25 mm; pronotal width across paranota 0.70–0.75 mm; hemelytral length 1.95–2.15 mm; maximum width of hemelytron 0.75–0.80 mm.

**Female.** General habitus very similar to that of male (Figs 2F, 4F, 6F, 8F, 10F, 13F) except for the following characters: body usually longer and wider than in male; antennal segments III and IV shorter than in male; hemelytron usually longer and wider than in male; and apical part of abdomen pentagonal in ventral view.

Measurements (*n* = 21). Body length with hemelytra 2.70–3.05 mm; maximum width of body across hemelytra 1.40–1.50 mm; length of antennal segments I to IV 0.20 mm, 0.10 mm, 1.00 mm, and 0.50 mm, respectively; pronotal length 1.15–1.30 mm; pronotal width across paranota 0.70–0.80 mm; hemelytral length 2.00–2.25 mm; maximum width of hemelytron 0.80–0.85 mm.

#### Remarks.

In previous studies (Souma and Kamitani 2021; Souma 2022a), *Omoplax kobugashi* sp. nov. was identified as *O. majorcarinae*, but the former differs from the original description and the illustrations of the latter (Guilbert 2001) in the following characters: body length with hemelytra 2.60–3.05 mm (3.45 mm in type material) (Fig. 1F, 2F); maximum width of body across hemelytra 1.20–1.50 mm (1.85 mm in type material); rostrum reaching posterior margin of mesosternum (Fig. 11F); pronotal disc black (Figs 3F, 4F, 5F, 6F); anterior margin of hemelytron weakly curved downward in apical half (Figs 7F, 8F, 9F, 10F); and Sc (subcosta) vein of hemelytron distinct in apical part of dorsal view. These features were considered an intraspecific variation of *O. majorcarinae* in previous studies (cf. Souma and Kamitani 2021; Souma 2022a), but constitute interspecific variation in the present study based on the examination of dozens of specimens which show two morphological species, each respectively collected from different plant species.

In general appearance, *Omoplax kobugashi* sp. nov. is very similar to *O. desecta*, whose distribution range in Chichijima Group is consistent with that of *O. kobugashi* sp. nov. (Souma and Kamitani 2021; Souma 2022a), but the former can be distinguished from the latter based on the rostrum reaching the posterior margin of the mesosternum (reaching the posterior margin of the metasternum in *O. desecta*) and the black pronotum (pale brown in *O. desecta*) (Figs 3B, F, 4B, F, 5B, F, 6B, 11B, F). Morphological differences between *O. kobugashi* sp. nov. and the five other *Omoplax* species are presented in the identification key below.

#### Distribution.

Japan: Ogasawara Islands: Chichijima Group (Anijima Island, Chichijima Island) (Fig. 19) (Souma and Kamitani 2021; Souma 2022a). *Omoplax kobugashi* sp. nov. is endemic to Chichijima Group.

#### Etymology.

The specific epithet is the Japanese plant name “Kobugashi” [= *Machilus kobu*], referring to the host plant of the new species; a noun in apposition.

#### Host plant.

Only *Machilus kobu* (Lauraceae) (Fig. 17H), which is also known as “Kobugashi”, was confirmed as a host plant for *Omoplax kobugashi* sp. nov. by the field and captive observations of adults and nymphs, suggesting the possibility of monophagy for this lace bug species.

#### Bionomics.

*Omoplax kobugashi* sp. nov. inhabits an evergreen broad-leaved forest with a subtropical climate in the Ogasawara Islands (Souma and Kamitani 2021), and sucks sap on the abaxial side of the leaves of *Machilus kobu*, causing irregular yellowing on the adaxial side (Fig. 17H). Adults were collected in September and from May to July (Souma and Kamitani 2021; Souma 2022a); nymphs were collected in September.

### ﻿ Omoplax majorcarinae

Taxon classificationAnimaliaHemipteraTingidae

Guilbert, 2001

F6716315-5CF9-5BB9-AC49-DCA87A0CFF90

Figs 1G, 2G, 3G, 4G, 5G, 6G, 7G, 8G, 9G, 10G, 11G, 12G, 13G, 14G, 16G–I

Omoplax majorcarinae Guilbert, 2001: 551. Holotype: ♂; type locality: Japan • Bonin Islands, Chichijima, Chuo san [= Ogasawara Islands, Chichijima Group, Chichijima Island, Mt. Chuosan]; Bernice P. Bishop Museum, Honolulu, Hawaii, USA.
Omoplax desecta (Horváth, 1912): Souma and Kamitani (2021: 8) (distribution: part); Souma (2022a: 125) (distribution: part); Shimamoto and Ishikawa (2023: 94) (catalog: part). Misidentifications.

#### References.

Yamada and Tomokuni (2012: 198) (monograph); Yamada and Ishikawa (2016: 432) (checklist: Japan); Shimamoto and Ishikawa (2023: 94) (catalog: part).

#### Material examined.

***Non-types***, Japan • 1 ♂; Ogasawara Isls., Ototojima Is.; 8 Jul. 1994; Y. Kaneko leg.; referring to Souma and Kamitani (2021); TUA • 3 ♂♂; Ogasawara Isls., Ototojima Is.; 2 Aug. 1996; T. Matsumoto leg.; referring to Souma (2022a); NSMT • 1 ♂; Ogasawara Isls., Anijima Is., Mt. Togari; 29 Jul. 2021; T. Matsumoto & S. Shimamoto leg.; SIHU • 1 ♀; Ogasawara Isls., Chichijima Is., Tsurihama; 10 Feb. 2024; Y. Hisasue leg.; SIHU • 1 ♀; Ogasawara Isls., Chichijima Is., Shigureyama; 9 Mar. 2024; Y. Hisasue leg.; SIHU • 2 ♂♂ 1 ♀; Ogasawara Isls., Chichijima Is., Mt. Mikazuki; 5 May 2024; N. Tsuji leg.; SIHU • 1 ♂; Ogasawara Isls, Chichijima Is., Mt. Mikazuki; 17 May 2024; Y. Hisasue leg.; SIHU • 1 ♀; same locality data as for preceding; 20 Aug. 2024; Y. Hisasue leg.; SIHU • 2 ♀♀; Ogasawara Isls., Ototojima Is., Ainosawa; 3–4 Jul. 2024; N. Tsuji leg.; SIHU • 1 ♂; Ogasawara Isls., Anijima Is., Mt. Maruyama; 9 Jul. 2024﻿; Y. Hisasue leg.; SIHU • 1 ♀; Ogasawara Isls., Ototojima Is., Mt. Sokuryogatake; 12 Jul. 2024; Y. Uehara leg.; SIHU • 1 ♀; Ogasawara Isls., Anijima Is., Mt. Omaru; 13 Jul. 2024; Y. Hisasue leg.; SIHU • 2 ♀♀; Ogasawara Isls., Chichijima Is., Hatsuneura; 28 Jul. 2024; N. Tsuji leg.; SIHU • 1 ♂; Ogasawara Isls., Chichijima Is., Mt. Yoake; 4 Aug. 2024; Y. Hisasue leg.; SIHU • 1 ♀; Ogasawara Isls., Chichijima Is., Mt. Nyuto; 10 Aug. 2024; Y. Hisasue leg.; SIHU • 2 ♀♀ 1 fifth instar nymph; Ogasawara Isls., Ototojima Is., Kurohama–Ichinotani; Neolitsea sericea var. aurata; 23 Sep. 2024; J. Souma leg.; SIHU • 3 ♂♂ 2 ♀♀; same locality, host plant, and collector data as for preceding; 5 Oct. 2024; SIHU • 2 ♂♂ 2 ♀♀; Ogasawara Isls., Anijima Is., Tamana Beach–Mt. Mikaeri; Neolitsea sericea var. aurata; 24 Sep. 2024; J. Souma leg.; SIHU • 2 ♂♂ 4 ♀♀; Ogasawara Isls, Ototojima Is., Shikahama–Mt. Hirone; 27 May 2025; S. Shimamoto leg.; SIHU • 5 ♂♂ 2 ♀♀; Ogasawara Isls, Nishijima Is.; 7 Jun. 2025; Y. Hisasue leg.; SIHU • 1 ♀; Ogasawara Isls, Ototojima Is., Ichinotani; 9 Jun. 2025; Y. Hisasue leg.; SIHU. The single nymph recorded above is in poor condition and is thus not described in the present study.

#### Diagnosis.

*Omoplax majorcarinae* is recognized among the other *Omoplax* species based on a combination of the following characteristics: rostrum reaching middle part of mesosternum (Fig. 11G); pronotal disc pale brown (Figs 3G, 4G, 5G, 6G); hood more than 0.5 times as wide as maximum width of head across compound eyes, not reaching apex of clypeus (Fig. 14G); paranotum without areolae in middle part, with areolae in remaining parts; anterior margin of hemelytron strongly curved downward in apical half (Figs 7G, 8G, 9G, 10G); subcostal and discoidal areas of hemelytron united; costal area narrower than fused subcostal and discoidal areas; Sc (subcosta) vein of hemelytron indistinct in apical part of dorsal view; R+M (fused radius and media) vein of hemelytron indistinct, not carinate; and ventral surface of body dark brown to black (Figs 12G, 13G).

#### Remarks.

The above specimens matched well with the original description and the illustrations of *Omoplax majorcarinae* (Guilbert 2001) in terms of their morphological characteristics, especially body size, coloration, rostral length, and the shape of the paranotum and hemelytron, which are not consistent with the specimens recorded as *O. majorcarinae* in the previous studies (Souma and Kamitani 2021; Souma 2022a): body length with hemelytra 3.10–3.45 mm (3.45 mm in type material) (Figs 1G, 2G); maximum width of body across hemelytra 1.65–1.95 mm (1.85 mm in type material); rostrum reaching middle part of mesosternum (Fig. 11G); pronotal disc pale brown (Figs 3G, 4G, 5G, 6G); paranotum without areolae in middle part, with areolae in remaining parts (Fig. 14G); anterior margin of hemelytron strongly curved downward in apical half (Figs 7G, 8G, 9G, 10G); and Sc (subcosta) vein of hemelytron indistinct in apical part of dorsal view. Therefore, the examined specimens were identified as *O. majorcarinae*. Morphological differences between *O. majorcarinae* and the six other *Omoplax* species are presented in the identification key below.

**Figure 18.**
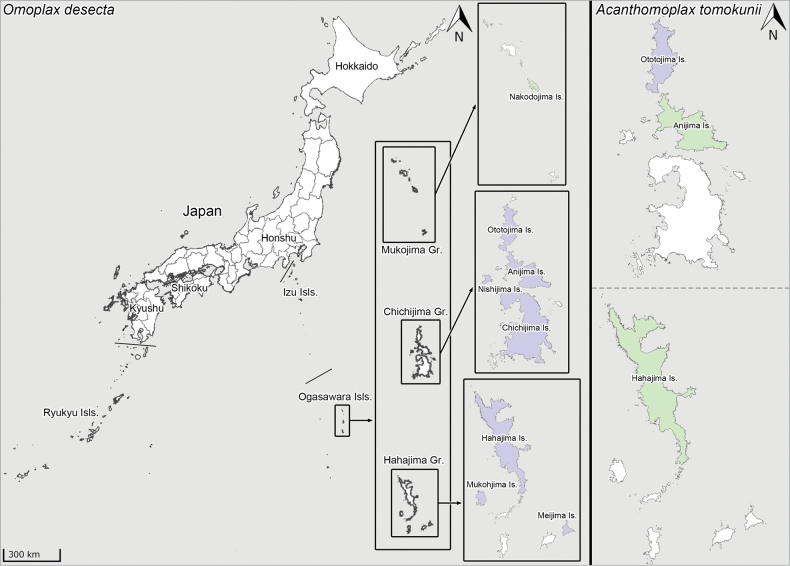
Collection sites of two tingid species endemic to the Ogasawara Islands, Japan. Blue-filled areas = localities based on the previous and present studies; green-filled areas = localities based on the previous studies only. The map was created with map data provided by the Geospatial Information Authority of Japan (GSI) (https://maps.gsi.go.jp).

#### Distribution.

**Figure 19.**
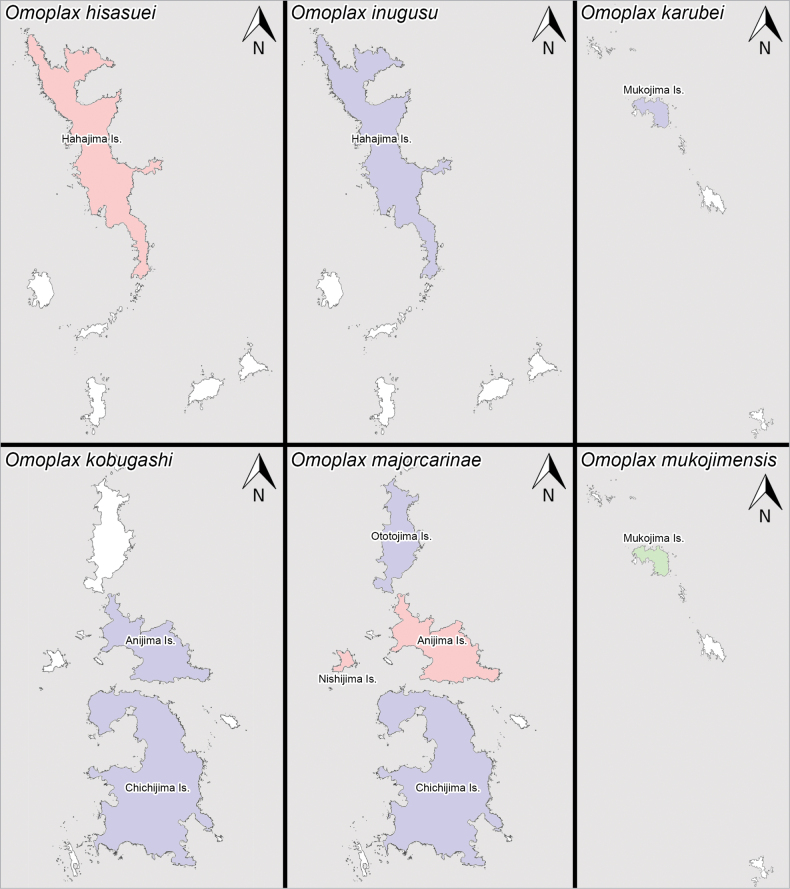
Collection sites of six tingid species endemic to the Ogasawara Islands, Japan. Red-filled areas = localities based on the present study only; blue-filled areas = localities based on the previous and present studies; green-filled areas = localities based on the previous studies only. The map was created with map data provided by the Geospatial Information Authority of Japan (GSI) (https://maps.gsi.go.jp).

Japan: Ogasawara Islands: Chichijima Group (Anijima Island, Chichijima Island, Nishijima Island, Ototojima Island) (Fig. 19) (Guilbert, 2001; Souma and Kamitani 2021; Souma 2022a). *Omoplax majorcarinae* is endemic to Chichijima Group and is newly recorded from Anijima and Nishijima islands.

#### Host plant.

Only Neolitsea sericea var. aurata (Lauraceae) (Fig. 17I), which is also known as “Kinshokudamo”, was confirmed as a host plant for *Omoplax majorcarinae* by the field and captive observations of adults and nymphs, suggesting the possibility of monophagy for this lace bug species. However, no feeding behavior of *O. majorcarinae* was observed on *Cinnamomum* sp. (Lauraceae) or *Ligustrum* sp. (Oleaceae), from which only a single adult was collected in a previous study (Guilbert 2001). Therefore, these two tree species do not appear to be host plants for this lace bug species.

#### Bionomics.

*Omoplax majorcarinae* inhabits an evergreen broad-leaved forest with a subtropical climate in the Ogasawara Islands (Souma and Kamitani 2021) and sucks sap on the abaxial side of the leaves of Neolitsea sericea var. aurata, causing irregular yellowing on the adaxial side (Fig. 17I). Adults were collected in February, March, and from May to October (Guilbert 2001; Souma and Kamitani 2021; Souma 2022a); a single nymph was collected in September.

### ﻿ Omoplax mukojimensis

Taxon classificationAnimaliaHemipteraTingidae

Souma, 2022

5189B309-9046-5C13-96EA-CE59DA41E20B

Figs 2H, 4H, 6H, 8H, 10H, 11H, 13H, 14H

Omoplax mukojimensis Souma, 2022a: 122. Holotype: ♀; type locality: Japan • “東京都小笠原村聟島南部” [= Ogasawara Islands, Mukojima Group, Muko­jima Island, Southern part]; KPMNH.

#### References.

Shimamoto and Ishikawa (2023: 94) (catalog); Souma (2023: 9) (monograph).

#### Material examined.

No additional specimens have been collected since the original description (Souma 2022a).

#### Diagnosis.

*Omoplax mukojimensis* is recognized among the other *Omoplax* species based on a combination of the following characteristics: rostrum reaching middle part of mesosternum (Fig. 11H); pronotal disc pale brown (Figs 4H, 6H); hood more than 0.5 times as wide as maximum width of head across compound eyes, not reaching apex of clypeus (Fig. 14H); paranotum with areolae throughout its length; anterior margin of hemelytron strongly curved downward in apical half (Figs 8H, 10H); subcostal and discoidal areas of hemelytron united; costal area narrower than fused subcostal and discoidal areas; Sc (subcosta) vein of hemelytron indistinct in apical part of dorsal view; R+M (fused radius and media) vein of hemelytron indistinct, not carinate; and ventral surface of body dark brown (Figs 13H).

#### Remarks.

Among all the *Omoplax* species, *O. mukojimensis* is most similar to *O. majorcarinae* in terms of its general habitus; however, the former can be distinguished from the latter based on the paranotum with areolae throughout its length (without areolae in the middle part and with areolae in the remaining parts in *O. majorcarinae*) (Figs 3G, 4G, H, 5G, 6G, H, 14G, H). Morphological differences between *O. mukojimensis* and the five other *Omoplax* species are presented in the identification key below.

#### Distribution.

Japan: Ogasawara Islands: Mukojima Group (Mukojima Island) (Fig. 19) (Souma 2022a). *Omoplax mukojimensis* is endemic to Mukojima Island.

#### Host plant.

Unknown (Souma 2022a).

#### Bionomics.

*Omoplax mukojimensis* inhabits an evergreen broad-leaved forest with a subtropical climate in the Ogasawara Islands (Souma 2022a). A single adult was collected in April; nymphs are unknown (Souma 2022a).

### ﻿Key to the species of Tingidae occurring in the Ogasawara Islands, Japan

Modified after the keys provided by Souma and Kamitani (2021) and Souma (2022a, 2023).

**Table:** 

1	Head with long spines (Figs 3A, 4A, 5A, 6A, 14A); paranotum narrowed posteriad; medial part of hood, median carina of pronotum, outer margin of paranotum, Sc (subcosta) and R+M (fused radius and media) veins of hemelytron with robust denticles throughout their respective length (Figs 7A, 8A, 9A, 10A)	***Acanthomoplax tomokunii* Souma & Kamitani, 2021**
–	Head with very short spines (Figs 3B–G, 4B–H, 5B–G, 6B–H, 14B–H); paranotum widened posteriad; hood, median carina of pronotum, outer margin of paranotum, Sc and R+M veins of hemelytron without robust denticles throughout their respective length (Figs 7B–G, 8B–H, 9B–G, 10B–H)	**2**
2	Rostrum reaching posterior margin of metasternum (Fig. 11B, E); ventral surface of body in various shades of brown (Figs 12B, E, 13B, E); feeding on Rhaphiolepis indica var. tashiroi (Rosaceae) or *Calophyllum inophyllum* (Clusiaceae)	**3**
–	Rostrum not reaching posterior margin of metasternum (Fig. 11C, D, F–H); ventral surface of body dark brown to black (Figs 12C, D, F, G, 13C, D, F–H); feeding on Lauraceae	**4**
3	Hood not reaching apex of clypeus (Figs 3B, 4B, 5B, 6B, 14B); paranotum without areolae in middle part, with areolae in remaining parts; anterior margin of hemelytron weakly curved downward in apical half (Figs 7B, 8B, 9B, 10B); R+M vein of hemelytron indistinct, not carinate; widespread in Ogasawara Islands, excluding Mukojima Island (Mukojima Group)	***Omoplax desecta* (Horváth, 1912)**
–	Hood reaching beyond apex of clypeus; paranotum with areolae throughout its length (Figs 3E, 4E, 5E, 6E, 14E); anterior margin of hemelytron not curved downward in apical half (Figs 7E, 8E, 9E, 10E); R+M vein of hemelytron distinct, carinate; endemic to Mukojima Island	***O. karubei* Souma, 2022**
4	Pronotal disc pale brown (Figs 3C, G, 4C, G, H, 5C, G, 6C, G, H); anterior margin of hemelytron strongly curved downward in apical half (Figs 7C, G, 8C, G, H, 9C, G, 10C, G, H); subcostal and discoidal areas of hemelytron united; Sc vein of hemelytron indistinct in apical part of dorsal view	**5**
–	Pronotal disc black (Figs 3D, F, 4D, F, 5D, F, 6D, F); anterior margin of hemelytron not or weakly curved downward in apical half (Figs 7D, F, 8D, F, 9D, F, 10D, F); subcostal and discoidal areas of hemelytron not united; Sc vein of hemelytron distinct in apical part of dorsal view	**7**
5	Paranotum without areolae in middle part, with areolae in remaining parts (Figs 3G, 4G, 5G, 6G, 14G); endemic to Chichijima Group	***O. majorcarinae* Guilbert, 2001**
–	Paranotum with areolae throughout its length (Figs 3C, 4C, H, 5C, 6C, H, 14C, H); endemic to Mukojima or Hahajima (Hahajima Group) islands	**6**
6	Hood less than 0.5 times as wide as maximum width of head across compound eyes (Figs 3C, 4C, 5C, 6C, 14C); costal area wider than fused subcostal and discoidal areas (Figs 7C, 8C, 9C, 10C); endemic to Hahajima Island	***O. hisasuei* sp. nov.**
–	Hood more than 0.5 times as wide as maximum width of head across compound eyes (Figs 4H, 6H, 14H); costal area narrower than fused subcostal and discoidal areas (Figs 8H, 10H); endemic to Mukojima Island	***O. mukojimensis* Souma, 2022**
7	Rostrum reaching middle part of mesosternum (Fig. 11D); paranotum with areolae throughout its length (Figs 3D, 4D, 5D, 6D, 14D); anterior margin of hemelytron not curved downward in apical half (Figs 7D, 8D, 9D, 10D); endemic to Hahajima Island	***O. inugusu* sp. nov.**
–	Rostrum reaching posterior margin of mesosternum (Fig. 11F); paranotum without areolae in middle part, with areolae in remaining parts (Figs 3F, 4F, 5F, 6F, 14F); anterior margin of hemelytron weakly curved downward in apical half (Figs 7F, 8F, 9F, 10F); endemic to Chichijima Group	***O. kobugashi* sp. nov.**

## ﻿Discussion

### ﻿Host plants and distribution ranges

In the present study, the host plants for seven of eight lace bug species from the Ogasawara Islands, excluding *O. mukojimensis*, were revealed based on field and captive observations. In addition, the distribution ranges of all eight species occurring in the Ogasawara Islands were clarified by the present and previous studies (e.g., Drake 1956; Guilbert 2001; Souma and Kamitani 2021; Souma 2022a). The present study provides basic knowledge for inferring the mechanisms of speciation of the endemic lace bugs from the Ogasawara Islands through evolutionary biological studies, with an allopatric distribution suggested depending on the host plants and island groups (islands). In the genus *Omoplax*, three pairs consisting of six species feeding on the same or congeneric host plants are allopatrically distributed in island groups or islands (Figs 17–19): (1) Of the two species feeding on the leaves of Rhaphiolepis indica var. tashiroi, one—*O. desecta*—is distributed on Nakodojima Island (Mukojima Group) and in Chichijima and Hahajima groups, and the other—*O. karubei*—is distributed only on Mukojima Island (Mukojima Group); (2) *O. hisasuei* sp. nov. and *O. majorcarinae* feed on the leaves of Neolitsea sericea var. aurata but are distributed on Hahajima Island (Hahajima Group) and in Chichijima Group, respectively; (3) *O. inugusu* sp. nov. and *O. kobugashi* sp. nov. feed on the leaves of *Machilus* species but are distributed on Hahajima Island and in Chichijima Group, respectively. Therefore, up to three *Omoplax* species are allopatrically distributed depending on host plant species in the same island groups or islands.

The two species in each of the three pairs of the genus *Omoplax* mentioned above possess morphological characteristics not observed in the other two pairs (Figs 1–13): (1) only *O. desecta* and *O. karubei* possess the rostrum reaching the posterior margin of the metasternum; (2) only *O. hisasuei* sp. nov. and *O. majorcarinae* possess the united hemelytral subcostal and discoidal areas, and the indistinct apical part of the hemelytral Sc (subcosta) vein in dorsal view; (3) only *O. inugusu* sp. nov. and *O. kobugashi* sp. nov. possess the black pronotal disc. Although further studies using molecular phylogenetic analyses are required, the similarities in morphological and ecological characteristics may reflect phylogenetic relationships within the genus *Omoplax*. In addition, *O. mukojimensis*, endemic to Mukojima Island, whose host plant is unknown, possesses the united hemelytral subcostal and discoidal areas and the indistinct apical part of the hemelytral Sc vein in dorsal view, similar to *O. hisasuei* sp. nov. and *O. majorcarinae*. The distribution ranges of *O. hisasuei* sp. nov., *O. majorcarinae*, and *O. mukojimensis* are consistent with the distribution of the genetic population of N. sericea var. aurata in the Ogasawara Islands (cf. Department of Forest Molecular Genetics and Biotechnology, Forestry and Forest Products Research Institute 2017). It is difficult to reveal a host plant for *O. mukojimensis* on Mukojima Island, which is uninhabited, has no nearby inhabited islands, and requires considerable time and expense to reach. However, field surveys focusing on N. sericea var. aurata may provide new information within a limited period. Moreover, the distribution ranges of *O. desecta* and *O. karubei* in Mukojima Group are consistent with the distribution of the genetic population of R. indica var. tashiroi (cf. Department of Forest Molecular Genetics and Biotechnology, Forestry and Forest Products Research Institute 2017). Further research on matches or mismatches in the spatial genetic structure of lace bugs and their host plants is needed to elucidate the mechanism of speciation in the genus *Omoplax*.

The only exception to the allopatric distribution of lace bugs in the Ogasawara Islands was *Acanthomoplax tomokunii*. Owing to its rarity, the information available on the host plant of *A. tomokunii* is only associated with the leaves of *Machilus kobu* on Ototojima Island (Chichijima Group). However, it is reasonable to assume that *A. tomokunii* feeds on *Machilus* species in other island groups or islands. *Acanthomoplax tomokunii* is sympatrically distributed on Anijima Island (Chichijima Group) with *O. kobugashi* sp. nov., which feeds on the leaves of *M. kobu*, and on Hahajima Island with *O. inugusu* sp. nov., which feeds on the leaves of a congener of *M. kobu* (*M. boninensis*). The remarkable morphological differences between the genera *Acanthomoplax* and Omoplax mentioned in the Results section suggest that A. tomokunii may be distantly related to *Omoplax* species and that interspecific hybridization may not occur even when inhabiting the same host species plant on the same island. However, further research is required to explain why *A. tomokunii*, which feeds on *M. kobu*, is sympatrically distributed on the two islands with two *Omoplax* species, which feed on the same or congeneric host plant.

In Japanese territories, excluding the Ogasawara Islands, of the 102 known lace bug species, ten species of the genus *Stephanitis* feed on lauraceous trees, and *Machilus thunbergii*, which is fed on by six species, is considered an important host plant (cf. Souma 2022b, 2023, 2024, 2025). In the Ogasawara Islands, where *Machilus thunbergii* is not distributed (cf. Tanaka and Matsui 2007–2025), at least five of the eight lace bug species feed on the lauraceous trees, and three species feed on endemic *Machilus* species (cf. Government of Japan 2010). Consequently, lauraceous trees, especially *Machilus* species, are considered important host plants for lace bugs from the Ogasawara Islands, similar to those from other regions of Japan. To estimate the origin of the endemic lace bug genera of the Ogasawara Islands, especially *Omoplax*, which was previously considered a subgenus of *Stephanitis* based on the similarity of their morphological characteristics (cf. Horváth 1912), further studies using molecular phylogenetics analyses, including allied taxa from other regions, such as Lauraceae-feeding species of *Stephanitis*, are required.

### ﻿Conservation of endemic lace bugs

The invasive green anole *Anolis carolinensis*, which threatens the endemic insects distributed in Chichijima (Anijima and Chichijima islands) and Hahajima (Hahajima Island) groups of the Ogasawara Islands, preys mainly on hemipterans and occasionally on *Omoplax desecta* (cf. Karube and Takakuwa 2004; Makihara et al. 2004; Karube 2005; Toda et al. 2009; Takahashi et al. 2014). The green anole control program in the Ogasawara Islands has been implemented by the Ministry of the Environment since 2005 (Toda et al. 2009). However, the risk of extinction for endemic lace bugs has not yet been assessed. To the best of the author’s knowledge, *O. desecta*, which is widespread in the Ogasawara Islands, is the most abundant endemic lace bug, occurring not only on Rhaphiolepis indica var. tashiroi and *Calophyllum inophyllum* growing wild in forests throughout most of its distribution range, including the islands where the green anole is not distributed, but also on *C. inophyllum* planted in urban areas (Figs 17C, D, 18). In addition to being easily found in almost all locations in the Ogasawara Islands, dozens of individuals were collected from Chichijima Island during field surveys in the 2020s, approximately 60 years after the invasion of the green anole (Toda et al. 2009), as shown in the Results section. Thus, the risk of extinction of *O. desecta* may be low. Among the other endemic lace bugs, *Acanthomoplax tomokunii*, *O. hisasuei* sp. nov., *O. inugusu* sp. nov., *O. kobugashi* sp. nov., and *O. majorcarinae* are distributed on Anijima, Chichijima, and/or Hahajima islands, where the green anole has invaded (Fig. 19) (Takahashi et al. 2014). These five species were rarely collected until their host plants were discovered in the 2020s. Therefore, their past distribution is unknown. The impact of green anole predation on the five lace bug species cannot be assessed with current knowledge, but continuous monitoring of the population status of *A. tomokunii* and *O. hisasuei* sp. nov., which appear to be rare, is necessary to conserve the endemic lace bugs: (1) *A. tomokunii* is distributed not only on Anijima and Hahajima islands, but also on Ototojima Island, where the green anole is not distributed, but even after conducting surveys focusing on the host plant (*Machilus kobu*), a few individuals were collected only from Ototojima Island; (2) because Neolitsea sericea var. aurata, on which *O. hisasuei* sp. nov. feeds, grows only in limited areas of Hahajima Island in Hahajima Group, *O. hisasuei* sp. nov., which is endemic to Hahajima Island, is unlikely to be distributed on other islands where the green anole has not been recorded; (3) in contrast, *O. inugusu* sp. nov., *O. kobugashi* sp. nov., and *O. majorcarinae* have been confirmed in several localities on the islands where the green anole has invaded in the 2020s, and considering the distribution of the host plants (*M. kobu*, *M. boninensis*, and N. sericea var. aurata), new localities may be discovered on islands where green anole is not distributed (cf. Government of Japan 2010; Takahashi et al. 2014). As with the lace bug, continuous monitoring of the population status of other phytophagous insects, especially endemic species whose host plants grow only on islands where the green anole has invaded, or whose localities are few, is necessary for conservation.

On the other hand, the invasive pink wax scale, *Ceroplastes rubens* Maskell, 1893 (Hemiptera, Sternorrhyncha, Coccidae), which is recorded from Chichijima and Hahajima groups in the Ogasawara Islands, feeds on the leaves of all host plants for the seven endemic lace bug species, namely, *M. kobu*, *M. boninensis*, and N. sericea var. aurata, R. indica var. tashiroi, and *C. inophyllum* (cf. Ota et al. 2024), raising the possibility of a competitive relationship between the pink wax scale and endemic lace bugs. Additionally, sooty mold induced by pink wax scale causes leaf drop and plant death (Kobayashi 1986; Ota et al. 2024), which may pose a future extinction risk to endemic lace bugs that feed on the same plants. The effects of pink wax scale and sooty mold on endemic phytophagous insects of the Ogasawara Islands, especially on folivorous species, are poorly understood but should be investigated in future studies for conservation purposes.

## Supplementary Material

XML Treatment for Acanthomoplax

XML Treatment for Acanthomoplax tomokunii

XML Treatment for Omoplax

XML Treatment for Omoplax desecta

XML Treatment for Omoplax hisasuei

XML Treatment for Omoplax inugusu

XML Treatment for Omoplax karubei

XML Treatment for Omoplax kobugashi

XML Treatment for Omoplax majorcarinae

XML Treatment for Omoplax mukojimensis

## ﻿Additional information

### Use of AI

No use of AI was reported.

### Author ORCIDs

Jun Souma https://orcid.org/0000-0002-2238-5015

### Data availability

All of the data that support the findings of this study are available in the main text.
